# Structure-Based Design of Selective Fat Mass and Obesity Associated Protein (FTO) Inhibitors

**DOI:** 10.1021/acs.jmedchem.1c01204

**Published:** 2021-11-11

**Authors:** Shifali Shishodia, Marina Demetriades, Dong Zhang, Nok Yin Tam, Pratheesh Maheswaran, Caitlin Clunie-O’Connor, Anthony Tumber, Ivanhoe K. H. Leung, Yi Min Ng, Thomas M. Leissing, Afaf H. El-Sagheer, Eidarus Salah, Tom Brown, Wei Shen Aik, Michael A. McDonough, Christopher J. Schofield

**Affiliations:** † The Chemistry Research Laboratory, Department of Chemistry and the Ineos Oxford Institute for Antimicrobial Research, University of Oxford, 12 Mansfield Road, Oxford OX1 3TA, U.K.; ‡ Department of Chemistry, Hong Kong Baptist University, Kowloon Tong, Hong Kong SAR 999077, China; § Chemistry Branch Department of Science and Mathematics, Suez University, Suez 43721, Egypt

## Abstract

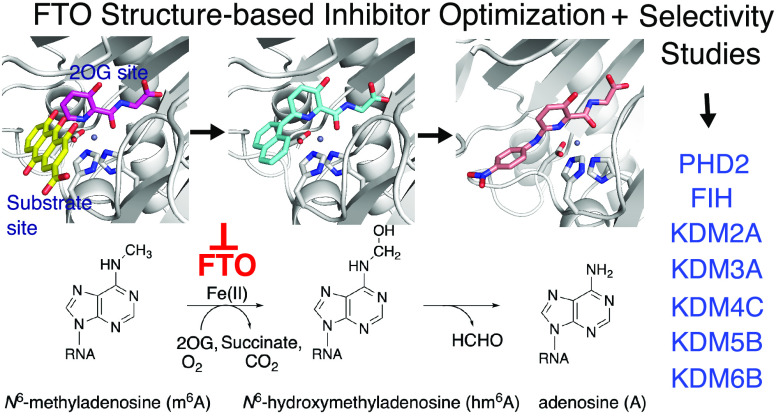

FTO catalyzes the Fe(II) and 2-oxoglutarate (2OG)-dependent modification of nucleic acids, including the demethylation of *N*^6^-methyladenosine (m^6^A) in mRNA. FTO is a proposed target for anti-cancer therapy. Using information from crystal structures of FTO in complex with 2OG and substrate mimics, we designed and synthesized two series of FTO inhibitors, which were characterized by turnover and binding assays, and by X-ray crystallography with FTO and the related bacterial enzyme AlkB. A potent inhibitor employing binding interactions spanning the FTO 2OG and substrate binding sites was identified. Selectivity over other clinically targeted 2OG oxygenases was demonstrated, including with respect to the hypoxia-inducible factor prolyl and asparaginyl hydroxylases (PHD2 and FIH) and selected JmjC histone demethylases (KDMs). The results illustrate how structure-based design can enable the identification of potent and selective 2OG oxygenase inhibitors and will be useful for the development of FTO inhibitors for use *in vivo*.

## Introduction

The fat mass and obesity associated protein (FTO) is an Fe(II) and 2-oxoglutarate (2OG)-dependent oxygenase that catalyzes the oxidation of *N*-methyl groups in nucleic acids, and, in particular, of *N*^6^-methyladenosine (m^6^A) in RNA.^1−3^ FTO is a potential medicinal chemistry target due to its roles in obesity and cancer. Single nucleotide polymorphisms in an intron of the *fto* gene were reported to be associated with body weight and type II diabetes in genome-wide association studies.^4,5^ More recent studies have reported roles for FTO in m^6^A regulation and cancers, including acute myeloid leukemia and glioblastoma.^6−11^ Precisely how RNA oxidation by FTO is linked to disease is uncertain. There is thus an unmet need to develop small-molecule probes to complement genetic studies and explore its potential as a therapeutic target.

**Figure 1:**
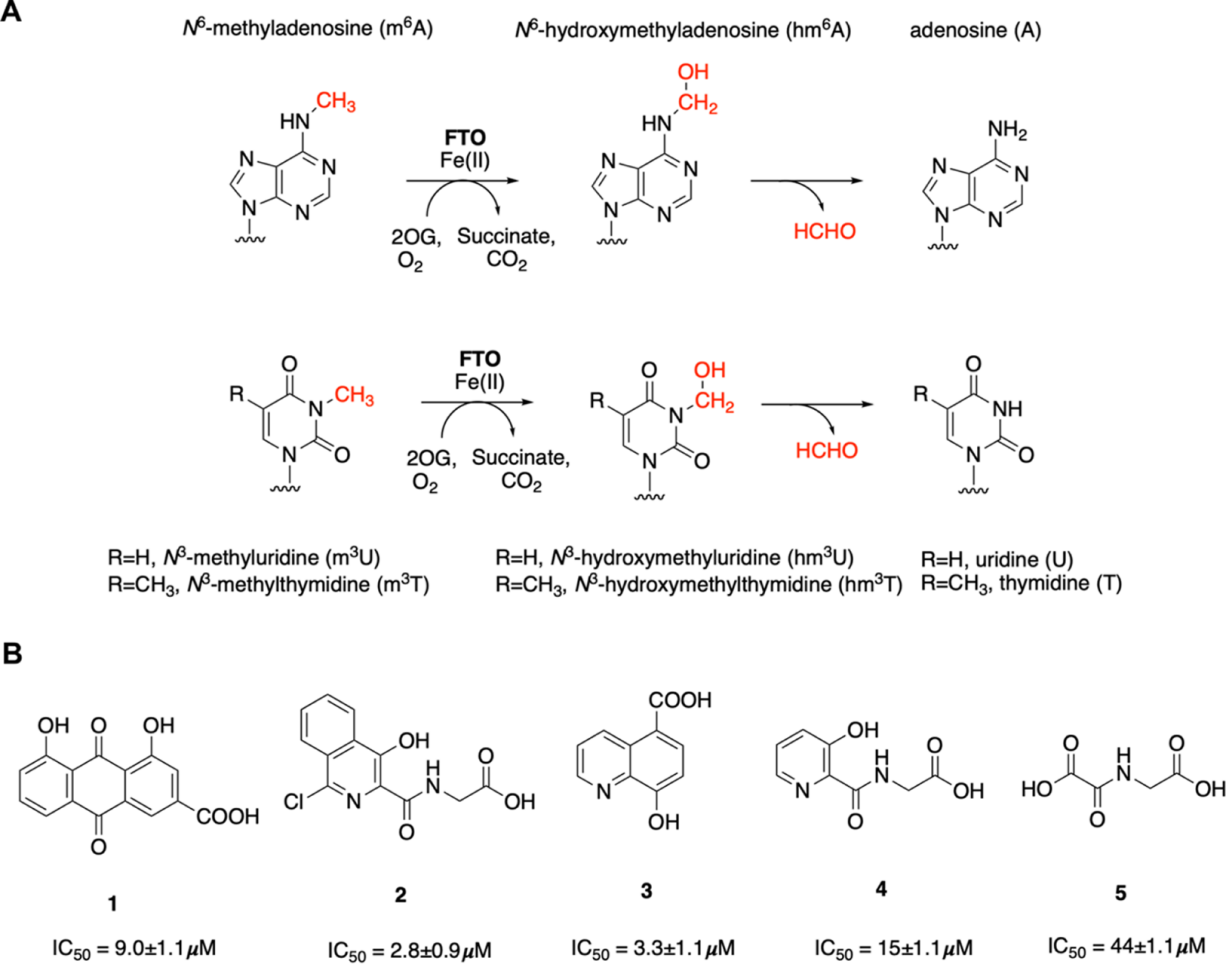
(A) Schematic representation of FTO-catalyzed reactions. (B) Reported FTO inhibitors with IC_50_^26^ values against FTO (determined using an LC assay with the m^3^T nucleoside as a substrate).^26^

FTO is one of 60–70 human 2OG oxygenases, some of which are medicinal chemistry targets, for example, the hypoxia-inducible factor prolyl hydroxylases (PHD 1–3) and selected JmjC *N*^ε^-methyllysine histone demethylases (KDMs).^12,13^ PHD inhibitors are approved for the treatment of anemia in chronic kidney disease, though there have been concerns regarding their safety.^14^ Human nucleic acid oxygenases (NAOXs), including FTO, are a substantial subset of 2OG oxygenases (>10 enzymes). NAOXs include eight AlkB homologues (ALKBH 1–8), some of which are involved in DNA damage repair (e.g., ALKBH2) and RNA oxidation (e.g., FTO and ALKBH5) and the Ten Eleven Translocation enzymes (TET1-3), which catalyze sequential oxidations of the methyl group of 5-methylcytosine.^15,16^ FTO catalyzes hydroxylation which can lead to demethylation of *N*-methyl groups on single-stranded RNA and DNA substrates (Figure 1A), including *N*^3^-methylthymidine (m^3^T), *N*^3^-methyluridine (m^3^U), m^6^A, and *N*^6^,2′O-dimethyladenosine (m^6^Am).^1−3,17^ The lifetime of the nascent *N*-hydroxymethylated products/intermediates varies depending on the nucleobase undergoing oxidation, leading to the proposal that in some circumstances they may be biologically relevant.^17−19^

There are some reports of FTO inhibition, including by known inhibitors of 2OG oxygenases, such as PHD inhibitors (Figure S1).^6,7,20−26^ We have reported crystallographic and *in vitro* inhibition studies of FTO with known 2OG oxygenase inhibitors including 2OG competitors/analogues (**2–4**) and rhein (**1**) (Figure 1B).^26,27^ The most potent FTO inhibitor identified, FG-2216 (**2**, IC_50_ 3 μM), is a PHD inhibitor.^28^ Treatment of mice with FG-2216 (**2**) led to reduced bone mineral density and altered adipose tissue density distribution without affecting body weight or respiratory exchange ratio.^29^ Structures of FTO in complex with **3** (FTO IC_50_ 3.3 μM) and **4** (FTO IC_50_ 15 μM) were also obtained.^26^**3** was reported as a broad spectrum inhibitor of the JmjC histone lysine demethylases (KDM2A, 3A, 4A-E, 5C, 6A, and B, 7B: IC_50_ 0.2–25 μM) and inhibits the bacterial NAOX AlkB (IC_50_ 3.4 μM).^26,30,31^ The reported FTO inhibitors thus likely do not have the potency or selectivity required for *in vivo* target validation of FTO. Here, we report the use of structural knowledge of how FTO binds 2OG and substrate to design potent and, at least partially selective, FTO inhibitors.

## Results

**Figure 2:**
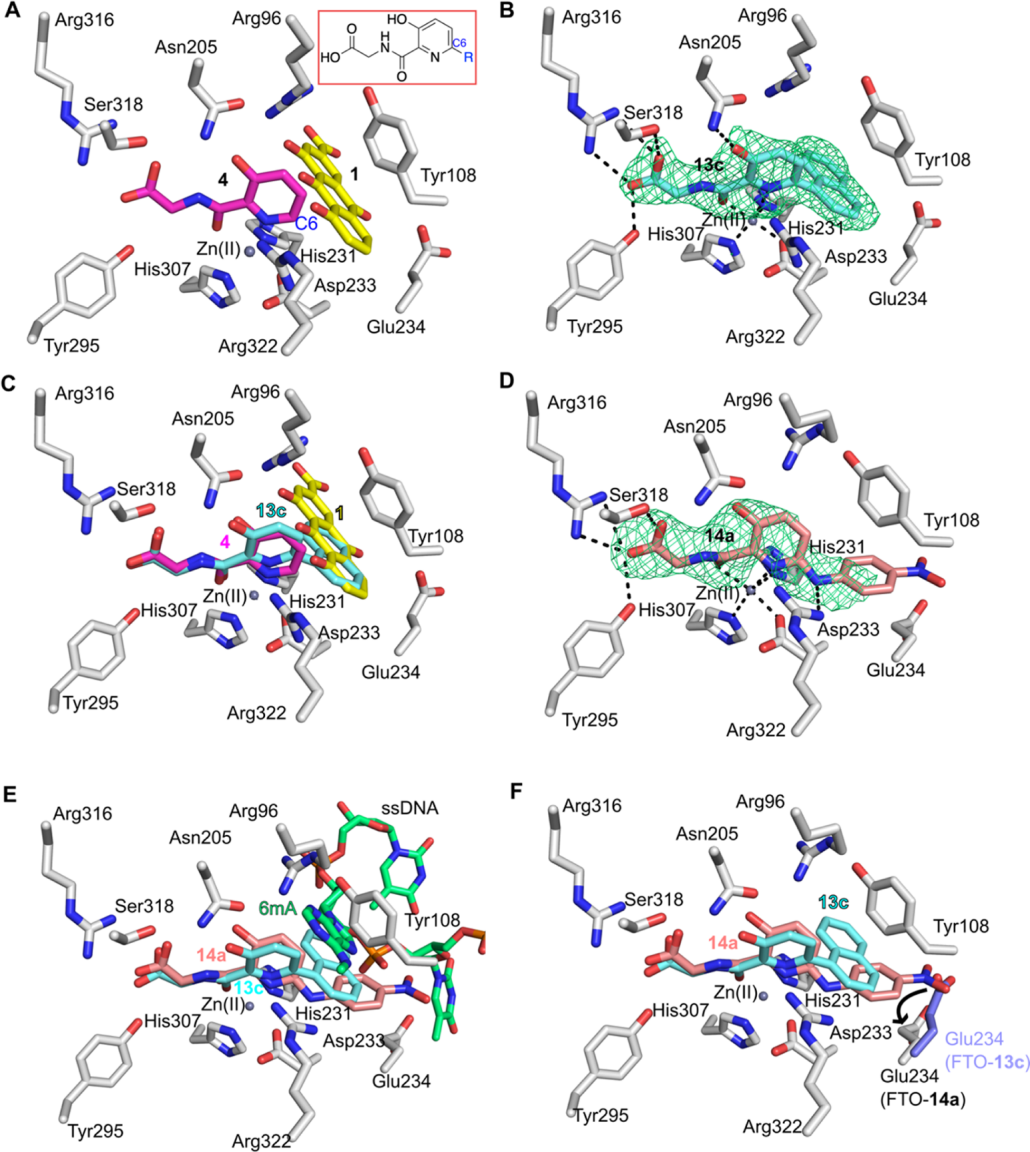
Structure-based FTO inhibitor design. (A) View of the active site from a crystal structure of FTOΔ31 (white sticks) in complex with **4** (magenta sticks) (PDB ID 4IE5),^26^ which was used as a basis for inhibitor design, with the structure of FTOΔ31-**1** (rhein, yellow sticks) (PDB ID 4IE7)^26^ superimposed. The inset indicates how the derivatization of **4** at C6 could enable inhibition by combining fragments of 2OG and nucleobase mimetics. (B) View of the active site from a structure of FTOΔ31 (white sticks) in complex with **13c** (cyan sticks surrounded by OMIT mF_o_-DF_c_ electron density displayed as green mesh, contoured to 3.0σ). (C) Superimposition of a structure of FTOΔ31-**13c** with that of FTOΔ31-**4** (PDB ID 4IE5; **4** in magenta) and FTOΔ31-**1** (PDB ID 4IE7, **1** in yellow). (D) View of the active site of FTO in complex with **14a**. The FTOΔ31 active site (white sticks) showing OMIT electron density (mF_o_-DF_c_, contoured to 3.0σ in a green mesh) around **14a** (salmon sticks). (E) View of superimposed structures of FTOΔ31-**13c** (PDB ID 4QHO, **13c** cyan sticks), FTOΔ31-**14a**, and a FTO-ssDNA substrate (lime green sticks) complex (PDB ID: 5ZMD). Only FTO residues (white sticks) from the FTOΔ31-**14a** structural complex are shown. (F) Superimposed structures of FTOΔ31-**13c** (PDB ID 4QHO, **13c** cyan sticks) and FTOΔ31-**14a**. Residues from FTOΔ31-**14a** are white sticks; the substrate binding Glu234 side chain from FTOΔ31-**13c** is highlighted in purple sticks. Note: the nitro-phenyl group of **14a** displaces the substrate binding Glu234 side chain. Non-carbon atoms colored: O (red), N (blue), Zn (gray sphere), and H-bond, electrostatic and metal coordination interactions are indicated as black dashes.

**Scheme 1:**
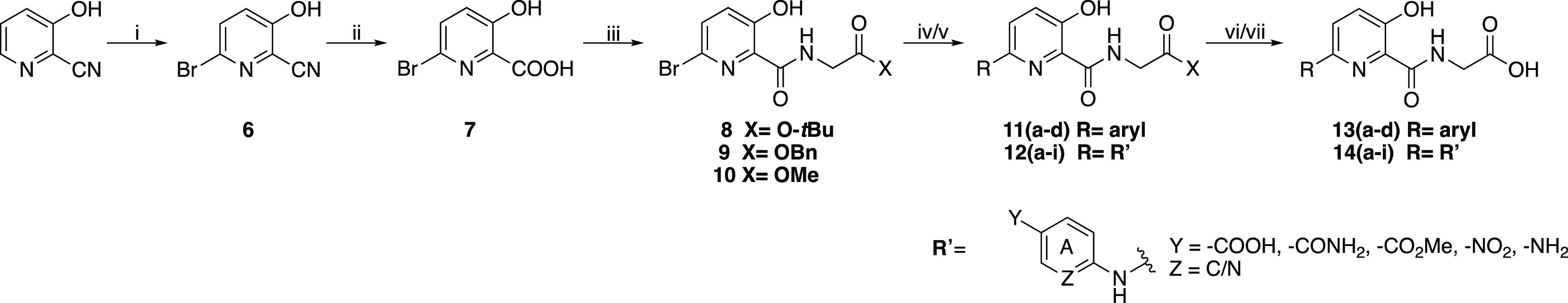
Synthesis of FTO inhibitors Suzuki or Buchwald–Hartwig Pd-couplings were used to couple the C-6 position of the pyridinyl ring with different aryl rings/aryl amines. Reagents and conditions: (i) Br_2_ (1.1 equiv), H_2_O, 3 days; (ii) NaOH 30%, MeOH, reflux, 12 h; (iii) GlyOC(CH_3_)_3_ or GlyOCH_3,_ or GlyOCH_2_Ph (1.5 equiv), propylphosphonic anhydride (1.5 equiv), diisopropylethylamine (4 equiv), microwave, 4 h; (iv) RB(OH)_2_ (1.3 equiv), K_2_CO_3_ (4 equiv), Pd(PPh_3_)_4_ (10 mol%), microwave, 130 °C; (v) ArNH_2_ (1.2 equiv), BrettPhos (0.15 equiv), BrettPhosLigand G1 (0.01 equiv), K_3_PO_4_ (1.2 equiv); (vi) KOH (excess), THF; (vii) Pd/C (10 mol%), THF, r.t.

During 2OG oxygenase catalysis, binding of 2OG in a bidentate manner to the active site Fe(II) is followed by that of the substrate, and then dioxygen.^12^ One strategy in 2OG oxygenase inhibitor design has been to covalently link a non-reactive 2OG mimetic with a group occupying the substrate binding site, so yielding more potent and selective inhibitors than the independent fragments.^32,33^ Unlike **2–5**, which bind in the 2OG binding pocket of FTO, rhein (**1**) occupies the nucleobase binding site.^26^ A comparison of the FTO and **4** (a glycyl-hydroxypyridine 2OG mimic) and FTO and rhein (**1**)^26^ structural complexes indicates that the hydroxypyridinyl ring of **4** is proximate to the anthraquinone ring of rhein (**1**), which binds competitively with the substrate nucleobase (Figures 2A and S2). Based on this analysis, we designed and synthesized a series of derivatives of **4** (Table 1) predicted to extend into the substrate binding site by functionalization at the C-6 position (**13a–d**) (Figure 2A, Scheme 1). 3-Hydroxypicolinonitrile was used as the starting material for C-6 derivatization of **4**. C-6 halogenation of the pyridine ring of hydroxypicolinonitrile, followed by hydrolysis of the nitrile gave 6-bromo-3-hydroxypicolinic acid (**7**), which was coupled with a protected glycine amide (glycine *tert*-butyl ester hydrochloride or glycine phenyl ester hydrochloride). The resulting compounds were coupled to different aryl rings by Suzuki or Buchwald couplings, then deprotected to give the desired acids (**13a**–**d** and **14a–i**).

**Table 1:**
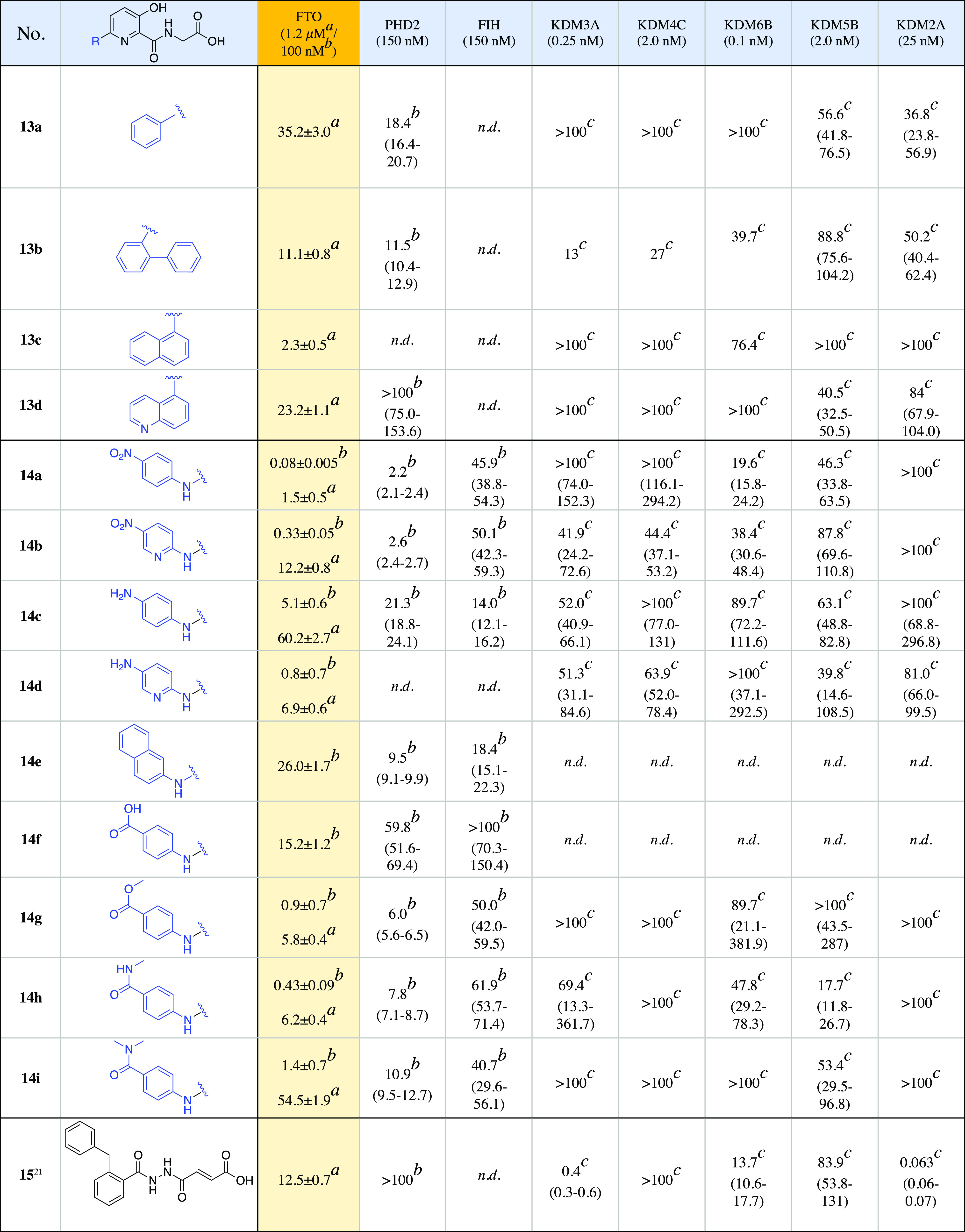
IC_50_ Values of Compounds against FTO and Selected Other Human 2OG Oxygenases

a IC_50_ values (in μM) obtained from the 5-mer m^6^A RNA LCMS-based demethylation assay.

b SPE-MS-based assay.^34^

c A histone demethylase luminescence-based AlphaScreen assay.^35^ For SPE-MS and AlphaScreen assays, the data represent IC_50_ values plus lower and upper 95% confidence limits (in parentheses). Enzyme concentrations employed are stated in parentheses on the first row. The high IC_50_ for **14c** (IC_50_ 5.1 μM) may in part be due its relative lack of solubility at the tested concentrations. Note: n.d., not determined.

We initially tested the *in vitro* inhibitory activity of **13a–d** against FTO (1.2 μM) using a reported liquid chromatography (LC)-based assay employing the m^3^T nucleoside as a substrate.^26^**13a** (IC_50_ 35 ± 1 μM), with a phenyl ring at C-6 was slightly less potent than **4** (IC_50_ 15 μM) against FTO. **13a** and **13b** (IC_50_ 15 ± 1 μM) had potencies similar to that of **4** against PHD2 and FTO; however, **13a** showed little or no inhibition (IC_50_ > 100 μM) toward the 2OG-dependent JmjC KDMs, KDM3A, KDM4C, and KDM6B and showed limited activity (IC_50_s 34–88 μM) toward KDM5A, B, C, and KDM2A (Tables 1 and S2). The introduction of a biphenyl ring at C-6 of the pyridine ring (**13b**) resulted in moderate FTO inhibition (IC_50_ 15 ± 1 μM), while manifesting an increased inhibition of the other tested 2OG oxygenases (PHD2, FIH, KDM3A, KDM4C, KDM6B, KDM5A, KDM5B, KDM5C, KDM5D, and KDM2A) compared to **13a** (Tables 1 and S2). Substitution at C-6 of the pyridine with 1-naphthalene (**13c**) gave more potent inhibition (IC_50_ 4 ± 1 μM), similar to that of FG-2216 (**2**) (IC_50_ 2.8 ± 0.9 μM). **13c** was, however, more selective for FTO, compared to **2** and the other compounds in this series over the JmjC KDMs. Quinoline derivative **13d** was prepared as a more basic and water-soluble analogue of **13c** but manifested a 6-fold decrease in potency against FTO (13d, IC_50_ 25 ± 1 μM) relative to **13c**.

To investigate the binding mode of **13c**, we obtained a structure of FTOΔ31 with Zn substituting for Fe in the complex with it (2.6 Å resolution, PDB ID 4QHO). The structure was solved by molecular replacement using an FTO-**4** complex structure (PDB ID 4IE5) as a search model. There was clear electron density for **13c** in the active site revealing that the hydroxypyridinyl glycyl scaffold manifests a similar binding mode to that previously observed for **4** (PDB ID 4IE5),^26^ with hydrogen bonding interactions occurring between the 3-hydroxy group on the pyridine ring and the Asn205 side chain and additional electrostatic and hydrogen bonding interactions between the glycyl carboxylate and the side chains of Arg316, Ser318, and Tyr295 (Figure 2B). As predicted, the **13c** 1-naphthyl side chain extends to occupy the substrate binding site (Figure 2B), with the plane of its naphthyl group being near orthogonal relative to its pyridine ring. The 1-naphthyl group of **13c** appears to form partial π–π interactions with the indole side chain of His231. The superimposition of the structures of FTOΔ31 in complex with **4**, **1**, and **13c**, show a substantial overlap, in particular the naphthyl group of **13c** is positioned similar to two of the three anthraquinone rings of rhein (**1**) (Figure 2C).

Because **13c** showed enhanced potency and higher selectivity compared to reported FTO inhibitors, we used the FTOΔ31-**13c** structure as a basis for the design of further inhibitors. Analyses of the FTOΔ31-**13c** structure suggests that the introduction of a heteroatom between the pyridinyl ring and the C-6 aryl substituent of the original series would provide additional side chain flexibility and potentially position the aryl ring between the side chains of Tyr106 and Tyr108.

Buchwald–Hartwig coupling was thus used to prepare the aniline derivatives (**14a–i**), which were tested against FTO and 10 other 2OG oxygenases (Tables 1 and S2). We initially employed an optimized LCMS-based assay using a 5-mer m^6^A RNA oligonucleotide as the substrate and 1.2 μM FTO (Figure S3) to investigate the IC_50_s of **13a–d**, **14a–d**, **14g–i**, and **15** (a reported FTO inhibitor).^21^ The IC_50_s of **14a–d** and **g–i** range from 1.5 to 60.2 μM, with the majority displaying increased potencies over **13a–d** (2.3–35.2 μM) (Table 1). The most potent of the tested FTO inhibitors was **14a** (IC_50_ 1.5 μM), which has a *p*-nitrophenyl side chain (Table 1). Against the panel of 2OG oxygenases, **14a–d** and **g–i** showed generally greater selectivity than **13a–d** toward FTO. For the majority of JmjC KDMs screened, IC_50_ values were in the tens of μM to >100 μM. The selectivity of the **14a–d** and **g–i** series for FTO over PHD2 was less pronounced (IC_50_ range 2.2–59.8 μM) but with the most potent FTO inhibitor, **14a**, being ∼30-fold more selective for FTO over PHD2 under the tested conditions.

With the LCMS-based assays, the IC_50_ measurement of **14a** (1.5 μM) was limited as it was near the FTO concentration (1.2 μM). We thus developed a more sensitive hydroxylation assay employing a lower FTO concentration (100 nM) with a solid-phase extraction coupled to MS (SPE-MS) assay (Figure S4, Table 1). Instead of demethylation, this assay monitors the hydroxylated product (hydroxymethyladenine, hm^6^A) of a 15-mer m^6^A-containing RNA oligonucleotide (hm^6^A has been reported to be a relatively stable adduct).^17,19^ We determined the IC_50_s of **14a–i** using SPE-MS.

The SPE-MS results show that the two compounds with *p*-nitrophenyl/*p*-nitropyridinyl groups at C-6 of the core pyridine, that is, **14a**/**14b**, are the most potent of the FTO inhibitors (IC_50_s 80 and 330 nM, respectively), followed by the *N*-methyl amide-phenyl (**14h**, IC_50_ 430 nM), amine-pyridyl (**14d**, 800 nM), ester (**14g**, 900 nM), *N*-dimethyl amide-phenyl (**14i**, 1.4 μM), and amine-phenyl (**14c**, IC_50_ = 5.1 μM) C-6 substituents. The compounds with benzoic acid **14f** and naphthylamine **14e** side chains were the weakest FTO inhibitors (IC_50_s 15.2 and 26 μM, respectively). Even though the C-6 nitro-pyridine analogue (**14b**) has a 4-fold higher IC_50_ than the nitro-phenyl analogue **14a**, the amine-pyridine substituent **14d** has a lower IC_50_ than the amine-phenyl derivative **14c**, thus pyridines at C-6 can be productively accommodated within the active site.

Changing the para-nitro group of the C-6 phenyl ring of **14a** to an amide, as in the monomethyl amide **14h** (IC_50_ 430 nM) or dimethyl amide **14i** (IC_50_ 1.4 μM) increases the IC_50_ values by ∼5–18 fold over **14a**, respectively. In general, substituting the nitro group of **14a** (amine: **14c** and **14d**; amide: **14h** and **14i**; carboxylate: **14f**; and ester: **14g**) resulted in a reduction in potency.

The results of testing **13a–d**, **14a–i**, and a previously reported selective FTO inhibitor **15**,^21^ against a panel of human 2OG oxygenases (Tables 1 and S2) revealed that, except for hydrazine compound **15** (FTO IC_50_ 12.5 ± 0.7 μM; LCMS-based assay), the hydroxy pyridine-glycyl compounds were for the most part selective for FTO and PHD2. Interestingly, **15** was identified as a potent inhibitor of the histone H3 lysine 36 JmjC KDM2A (IC_50_ 63 nM), which is a potential cancer target.^36,37^

To confirm that the compounds inhibit FTO by binding at the active site, we performed a competition-based NMR binding assay^38^ (using 2OG as a competitor) to obtain binding constants for the selected compounds (**13c**, **14a**, **14h**, and **14i**) (Figures S5 and S6). The rankings of the *K*_D_ values were generally in accord with the rankings of the IC_50_ values: **14a** (*K*_D_ 0.34 ± 0.16 μM, IC_50_ 1.5 ± 0.5 μM), **13c** (*K*_D_ 1.2 ± 0.24 μM, IC_50_ 2.3 ± 0.5 μM), **14h** (*K*_D_ 0.67 ± 0.29 μM, IC_50_ 6.2 ± 0.4 μM), and **14i** (*K*_D_ 3.9 ± 1.6 μM, IC_50_ 54.5 ± 1.9 μM).

We co-crystallized FTOΔ31 with **14a** and determined its structure (2.6 Å resolution). The structure revealed that the hydroxypyridyl-glycine core of **14a** binds, as observed for **4**, in the 2OG binding site (Figures 2D and S7). **14a** coordinates to the metal (Zn substituting for Fe) via its pyridinyl nitrogen and the carbonyl oxygen, its glycyl carboxylate forms a salt bridge with the side chain of Arg316 and hydrogen bonds with the side chains of Ser318 and Tyr295. In the FTOΔ31-**14a** complex, the **14a** carbonyl oxygen is positioned *trans* to the metal-coordinating residue Asp233 and the metal-coordinating pyridine nitrogen atom is *trans* to the metal coordinating residue His307. The plane formed by the pyridinyl ring of **14a** is skewed with respect to the plane formed by its pyridinyl nitrogen and carbonyl oxygen and the active site metal (Figure S7A,B). This orientation is unusual, especially when compared to the more typical co-planar orientation seen in the FTOΔ31-**4** structure (Figure S7B).

**Figure 3:**
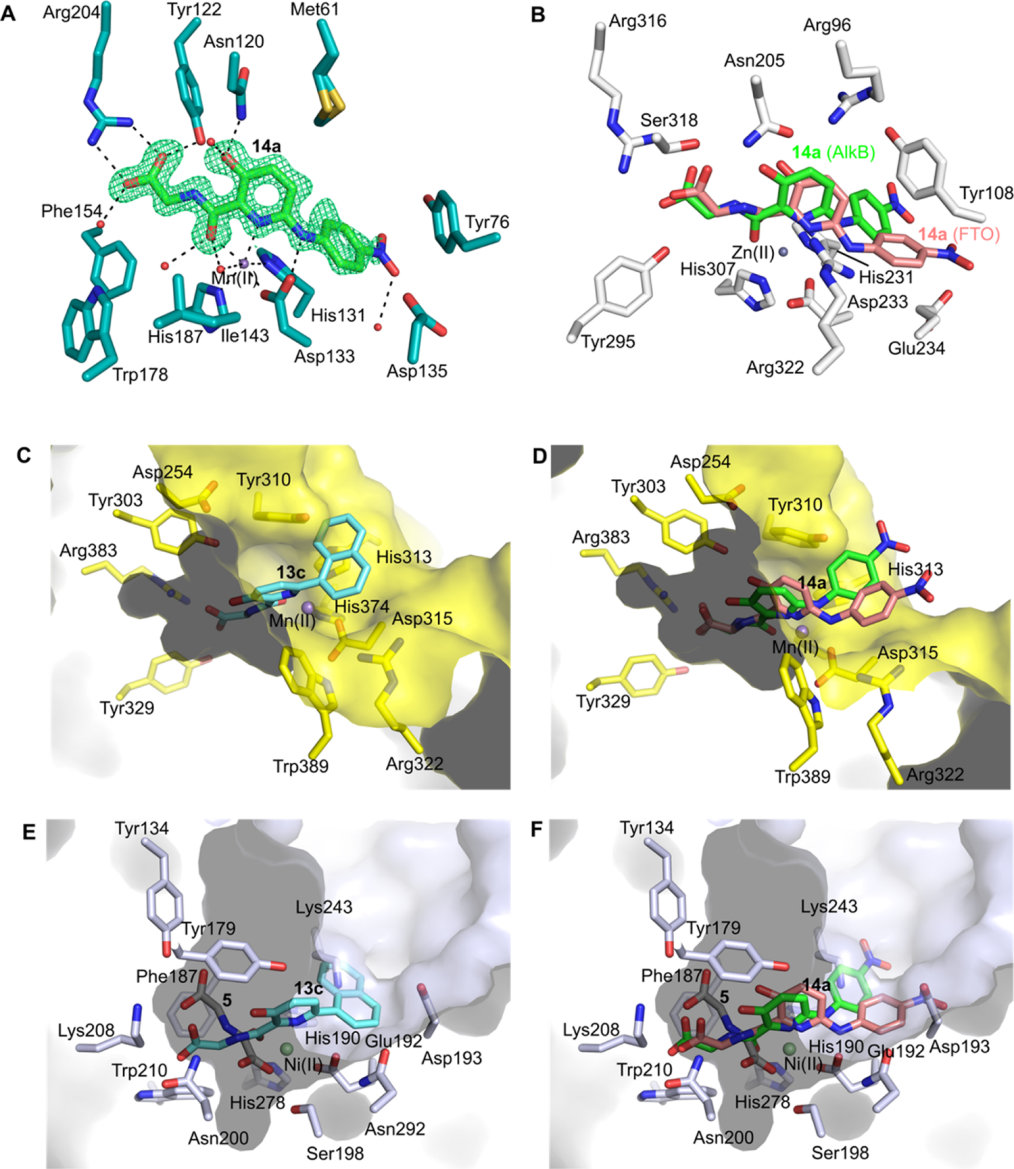
Structure-based rationalization of selectivity of FTO inhibitors. (A) View of the active site of the AlkBΔN11-**14a** complex (PDB ID 7NRO); **14a** (green sticks) with surrounding OMIT electron density mF_o_-DF_c_ shown as a green mesh and contoured to 3.0σ. (B) View of the FTOΔ31-**14a** structural complex (FTO in white sticks) (PDB ID 7E8Z) superimposed on a structure of AlkBΔN11-**14a**. Note: **14a** (salmon sticks, FTO; green sticks, AlkB) binds slightly differently to FTO and AlKB in particular, the metal-coordinating atoms of **14a** and the metal ion are co-planar with AlkB. (C) View of the active site from a structure of truncated PHD2 (yellow sticks and surface) superimposed with a structure of FTOΔ31 in complex with **13c** (PDB ID 4QHO) (derived from a structure of the PHD2-**2** complex PDB ID 4BQX,^39^ ligand not shown for clarity) indicating how the naphthyl side chain of **13c** may clash with Tyr310 in the PHD2 active site, so reducing is affinity (FTOΔ31 residues are not shown for clarity). (D) View of the active sites of structures of FTOΔ31 and AlkBΔN11 in complex with **14a** (FTO, salmon sticks; AlkB, green sticks) (PDB ID 7E8Z and 7NRO) superimposed onto PHD2 (yellow sticks and surface). Note the nitrophenyl side chain of **14a** is predicted to occupy the PHD2 active site opening with a potential for minor clashes, notably for the crystallographically observed AlkB **14a** binding mode (green sticks). (E) View from a structure of FTO in complex with **13c** superimposed onto a structure of KDM4C in complex with **5** (PDB ID 2XML)^40^ (KDM4 C shown as light blue sticks and surface; **5** in gray sticks) showing that the side chain of **13c** may clash with Lys243 of KDM4C. (F) View from complexes of FTOΔ31-**14a** (salmon sticks) and AlkBΔN11-**14a** (green sticks) superimposed on the structural complex of KDM4C-**5** (PDB ID 2XML)^40^ showing how the C-6 napthyl side chain of **14a** may clash with Lys243 and/or Asp193 of KDM4C. Non-carbon atoms colored: O (red), N (blue), Zn (gray sphere), Mn (purple sphere), and Ni (light green); H-bond, electrostatic, and metal coordination interactions are indicated as black dashes.

To investigate the generality of the skewed binding mode of **14a** as observed with FTO, we solved a crystal structure of a truncated version of the related bacterial NAOX AlkB (AlkBΔN11) in complex with it (Figure 3A). By contrast with the crystallographically observed FTO binding mode of **14a**, in the AlkB-**14a** structure, the metal-coordinating atoms of **14a** and the metal-ion are co-planar with the pyridinyl ring (Figure 3B). Contrary to the binding mode observed with FTO, the *p*-nitrophenyl group of **14a** is directed way from the center of the AlkB active site without significantly displacing key substrate binding side chains (Figure 3A,B). The binding mode of **14a** in AlkB is different from the previously reported potent AlkB glycinamide inhibitors where their side chains are typically positioned in a hydrophobic pocket close to Trp178, Ile143, and Phe154 (Figure S8).^31^

As predicted, the amine linker of **14a** apparently enables its *p*-nitrophenyl group to have a different orientation in FTO compared to the orientation of the naphthalene of **13c** (Figure 2F). With **14a**, the nitrogen of the amine linker is positioned to potentially form a hydrogen bond with the Arg322 side chain (2.9 Å); the Arg322 side chain is also positioned to form a cation−π interaction with the pyridine ring of **14a**. The *p*-nitrophenyl group of **14a** is positioned between the Glu234 and Tyr108 side chains of FTO. Notably, the Glu234 side chain is displaced ∼5 Å by the *p*-nitrophenyl moiety toward the surface of the protein (Figures 2F and S9), an unprecedented observation in available FTO inhibitor structures (Figure 2).^7,20,21,23−26,41^ Glu234 is important as it has also been shown to interact with m^6^A in a structure of an FTO variant in complex with a ssDNA substrate.^42^ Analysis of the FTO variant:ssDNA structural complex suggests that electrostatic repulsion between the negatively charged Glu234 side chain and the phenyl carboxylate of **14f** (IC_50_ 15.2 μM) may account for the large (190-fold) difference in IC_50_ between the isosteres **14a** and **14f**. Removing the inhibitor carboxylate group by substitution with a methyl ester **14g** (IC_50_ 860 nM) appeared to partially rescue potency resulting in a 10-fold difference relative to **14a**. The structural analysis implies that bulky naphthalene of **14e** would encounter steric hindrance in the pocket of FTO where the *p*-nitrophenyl group of **14a** is situated, explaining its significant 325-fold lower potency (IC_50_ 26 μM).

## Discussion and Conclusions

Our structure-guided approach to FTO inhibition was based on the knowledge that although Fe(II) and 2OG binding by 2OG oxygenases occurs by a conserved general process, that of substrate binding varies considerably, not least because the nature of the substrates varies for different sub-families of human 2OG oxygenases.^12,15^ Following analysis of structures of FTO in complex with a 2OG mimic (**4**) and rhein (**1**), which binds competitively with the substrate nucleobase (Figure S2), this approach resulted in compounds (e.g., **13b** and **c**) which were more potent than **4**. Following crystallographic studies of FTO with **13c**, derivatives of **4** were further optimized to highly potent FTO inhibitors such as **14a**, the proposed general binding mode of which was also validated by crystallographic analyses. The 2OG-competing inhibition mode of these inhibitors was supported by NMR studies.

Importantly, although there is likely scope for further optimization using a larger set of 2OG oxygenases in counter screening, some of the compounds we developed show selectivity for FTO (Tables 1 and S2), which in some cases can be rationalized on the basis of structural information. For instance, the set of compounds with an aryl ring directly linked to C-6 of the pyridine ring of the scaffold (**13a–d**) are generally more selective for FTO over PHD2, compared to **14a–i** where the aryl ring is linked to the pyridine C-6 via an amine. The superimposition of our FTOΔ31-**13c** structural complex with that of PHD2-**2**^39^ reveals that the C-6 aryl ring of **13c** will likely clash with the PHD2 Tyr310 side chain (Figure 3C), thus rationalizing in part why this set of FTO inhibitors is selective toward FTO over PHD2. In contrast, the superimposition of the FTOΔ31-**14a** and AlkB-**14a** structures with the PHD2-**2** structure reveals that the nitrophenyl group of **14a** will likely project into an unoccupied space at the active site entrance of PHD2 (Figure 3D). The lack of steric hindrance for the C-6 aryl ring of **14a** (and by implication) when modeled in PHD2 is thus proposed to lower its selectivity toward FTO over PHD2. The superimposition of the structural complexes of FTOΔ31-**13c**, FTOΔ31-**14a**, and AlkBΔN11-**14a** with KDM4C-**5** as a representative of JmjC oxygenases suggests that similar steric clashes of **14a** proposed for PHD2 may also occur in the KDM4C active site (Figure 3E,F). The superimposed structures also reveal that the hydroxypyridinyl compounds adopt a different metal-ion binding mode as **5** does with KDM4C, potentially providing a structural explanation for the selectivity of the C-6 substituted hydroxypyridine compounds for FTO over the JmjC sub-family of 2OG oxygenases.

It should, however, be noted that apparently clear crystallographically based structural explanations for the selectivity of inhibitors for particular 2OG oxygenases do not always manifest in experimental studies, as shown by the results with PHD inhibitors^43−46^ and some of our results for JmjC KDM inhibition (Tables 1 and S2). In this regard, it is important to note that, both in terms of 2OG and substrate binding by 2OG oxygenases, there is substantial evidence for conformational changes, as exemplified with PHD2, where such changes help enable a mechanism employing an ordered sequential active site binding of 2OG, substrate, and then dioxygen.^43,44^ Such conformational changes, at least in some cases, are observed on inhibitor binding, as observed with the PHDs.^45,46^ Further, with the JmjC KDMs, in some cases there is evidence for the inhibitor-induced Fe(II) movement.^30^ In the case of FTO, our structure with **14a**, provides evidence that the side chain of Glu234, which is likely important in catalysis, is susceptible to conformational changes (Figures 2F and S8). In the case of the structure of the complex of FTOΔ31-**14a**, a skewed inhibitor binding mode was observed; the skewed binding was less apparent in a structure of the bacterial NAOX AlkBΔN11 in complex with **14a**. Although further detailed work is required to define the precise binding mode of **14a** to FTO in solution (perhaps employing NMR), our observations illustrate why the crystallographic information should be used with care and highlight the importance of solution and empirical inhibition studies.

Nonetheless, the combined results illustrate how the knowledge of the 2OG cosubstrate and prime substrate binding modes as revealed by high-resolution crystallographic analyses can be combined to develop potent inhibitors of FTO, which is a human 2OG oxygenase of current therapeutic interest. Future work can now be directed toward the optimization of the compounds described here for *in vivo* studies aimed at defining how the reactions catalyzed by FTO are linked to its roles in physiology and disease.

## Experimental Section

### Recombinant FTO and FTOΔ31 Production and Purification

The production and purification of FTO and FTOΔ31 were carried out as reported.^26^*Escherichia coli* BL21 (DE3) cells (or *E. coli* Rosetta (DE3) pLysS cells for FTOΔ31-**14a**) were transformed with the pET28a_FTO plasmid (encoding for N-terminally hexahistidine-tagged full-length human FTO without the thrombin cleavage site between the hexahistidine tag and the protein) or pET28a_FTO FTOΔ31 (encoding for N-terminally hexahistidine-tagged human FTO with residues 2–31 deleted from the N-terminus) and were grown (37 °C; 180 rpm) to an OD_600_ of 0.6–0.8. FTO production was induced by the addition of a final concentration of 0.5 mM isopropyl β-d-1-thiogalactopyranoside (IPTG); growth was continued at 16 °C for 16 h (FTO) or 18 °C for 8 h (FTOΔ31). The resultant cell pellets were stored at −80 °C. The cell pellets were thawed and resuspended in 20 mM Tris, pH 7.5; 500 mM NaCl; 10 mM imidazole, and 1 mg of DNaseI, 10 mM MgCl_2_, and a Roche cOmplete protease inhibitor tablet (or 17.8 μg/mL phenylmethylsulfonyl fluoride for FTOΔ31-**14a**) then lysed by sonication on ice. The lysates were cleared by centrifugation, and the supernatant was loaded onto a 5 mL HisTrap column (Cytiva) and purified using an AKTA FPLC system. The column was treated with 20 mM Tris, pH 7.5; 500 mM NaCl; and 40 mM imidazole, then eluted with 20 mM Tris, pH 7.5; 500 mM NaCl; and 500 mM imidazole. The FTO solution was then buffer-exchanged into 25 mM Tris, pH 7.5, and concentrated to 20–40 mg/mL for storage. For crystallographic studies, the eluted FTOΔ31 from the HisTrap column was treated with EDTA (final concentration 200 mM) and incubated on ice for 30 min. The FTOΔ31 solution was desalted using a PD-10 desalting column with buffer A (25 mM Tris, pH 7.5), then further purified by a 5 mL HiTrap Heparin column (Cytiva), followed by a MonoQ column (Cytiva); both columns were equilibrated with buffer A (25 mM Tris–HCl, pH7.5) and eluted with a gradient from 0 to 100% buffer B (25 mM Tris, pH7.5; 1 M NaCl) for heparin chromatography and 0 to 60% buffer B for MonoQ ion exchange chromatography. The purified protein was then buffer-exchanged into buffer A and concentrated to 20 mg/mL for FTOΔ31-**13c** and 36 mg/mL for FTOΔ31-**14a**. The protein was snap frozen in liquid nitrogen and stored at −80 °C.

### Purification of the FTOΔ31-**14a** Complex

A final concentration of 8 mg/mL of FTOΔ31 was mixed with a final concentration of 1 mM ZnSO_4_ and a final concentration of 1 mM **14a** in 25 mM Tris, pH 7.5, then incubated on ice for 30 min. The solution was loaded onto a Superdex 200 increase 10/300 GL column (Cytiva), pre-equilibrated with 25 mM Tris, pH 7.5, for purification using size exclusion chromatography. Relevant fractions corresponding to the FTOΔ**31**-**14a** complex were pooled and concentrated to 10 mg/mL.

### AlkBΔN11 Expression and Purification for Crystallographic Studies

AlkBΔN11 was produced and purified as described.^31^ In brief, *E. coli* BL21 (DE3) cells were transformed with pET24a_AlkBΔN11 and allowed to grow at 37 °C until the OD_600_ reached 0.6 at which point AlkBΔN11 production was induced with a final concentration of 0.5 mM IPTG. The cells were incubated at 18 °C, 180 rpm overnight (ca. 18 h). Cells pellets were thawed and resuspended to homogeneity in 0.1 M MES pH 5.8 containing 1 mM MgCl_2_ with 1 mg of DNaseI and Roche cOmplete EDTA-free protease inhibitor cocktail. Cells were lysed by sonication on ice and the supernatant was obtained by centrifugation. The cell lysates were purified by cation exchange chromatography using a 50 mL S Sepharose column, with elution achieved by application of a gradient to 0.1 M MES buffer, 1 M NaCl, pH 5.8. The eluted protein was further purified using a 300 mL Superdex 75 column (Cytiva), pre-equilibrated in 50 mM HEPES, pH 7.5.

### *In Vitro* Demethylation/Hydroxylation Assays for FTO

#### LC-Based Assays Using the m^3^T Nucleoside

LC-based *in vitro* m^3^T demethylation assays were performed as reported.^26^ For initial assays using LC with the m^3^T nucleoside substrate, a 50 μL of reaction mixture containing final concentrations of 3 μM FTO; 70 μM m^3^T nucleoside; 160 μM 2OG; 500 μM l-ascorbate; 100 μM diammonium iron(II) sulfate complex; 200 μM compound (or DMSO alone in control reactions); and 50 mM 2-(*N*-morpholino)ethanesulfonic acid (MES), pH 6.3, was incubated at room temperature for 1 h. Reactions were quenched with methanol (50 μL), then centrifuged to remove the precipitated protein. The supernatant was then dried using an Eppendorf Speedvac concentrator and reconstituted with (50 μL) water. The product (thymidine) and substrate (m^3^T) were separated using a Waters Acquity Ultra Performance LC (UPLC) BEH C18 column (130 Å, 1.7 μm, 2.1 mm × 50 mm) with a gradient of 95% A (H_2_O with 1% formic acid) to 80% B (methanol with 0.1% formic acid) over 5 min. The UV detection wavelength was set to 266 nm. Elution was performed at room temperature; thymidine = 0.95 min, m^3^T = 1.48 min. The peaks were identified in the positive ion mode using electrospray ionization-time-of-flight (ESI-TOF) mass spectrometry (Waters LCT Premier XE machine). UV peaks were integrated using MassLynx software (Agilent), and the percentage conversion of m^3^T to thymidine was used to quantify the activity of FTO in the presence of inhibitors. For IC_50_ determinations, assays were carried out in triplicate with a range of compound concentrations (0, 10, 30, 100, and 300 μM and 1, 3, and 10 mM). IC_50_ values were calculated from dose–response curves plotted using GraphPad Prism 5.

### LCMS-Based Assay Using the RNA m^6^A 5-Mer Oligonucleotide

For assays using LC mass spectrometry (LC-MS) with a 5-mer ssRNA oligonucleotide substrate [GG(m^6^A)CU] (Ella Biotech, Germany), a 25 μL of reaction mixture containing final concentrations of 1.2 μM FTO; 10 μM 5-mer ssRNA oligonucleotide [GG(m^6^A)CU]; 10 μM 2OG; 500 μM l-ascorbic acid; 10 μM diammonium iron(II) sulfate; and 25 mM Tris-HCl, pH 7.5, was incubated at room temperature for 30 min. After incubation, the reaction mixture was quenched with methanol (25 μL), then centrifuged (14,000 rpm, 10 min) in Seahorse polypropylene 96-well filter microplates with a 30 KDa molecular weight cutoff polyethersulfone membrane to remove FTO. The product [GGACU] and substrate [GG(m^6^A)CU] were separated using a Waters Acquity UPLC Oligonucleotide BEH C-18 column (130 Å, 1.7 μm, 2.1 mm × 50 mm) with a gradient of 98% buffer A to 70% buffer B over 8 min, at room temperature. 1,1,1,3,3,3-Hexafluoro-2-propanol (HFIP) and triethylamine were used as an ion-pairing buffer for better separation of the 5-mer oligonucleotide substrate [GGm^6^ACU] and the demethylated product [GGACU]. Buffer A: 200mM HFIP, 8.15 mM TEA buffer, 5% methanol, buffer B: 20% buffer A + 80% methanol. Separation of oligonucleotides was monitored by UV; the detection wavelength was 265–292 nm. Retention times for [GGACU] and [GG(m^6^A)CU] were 2.5 and 3.0 min, respectively. The masses for each peak were confirmed by ESI-TOF mass spectrometry (Bruker Daltonics, microTOF). The area under for the substrate ([GG(m^6^A)CU]) and product ([GGACU]) peaks were integrated using data analysis software (Bruker) in the negative ion mode, and the percentage conversion of [GG(m^6^A)CU] to [GGACU] was used to quantify the demethylation activity of FTO in the presence of inhibitors. For IC_50_ determinations, assays were carried out in triplicate with a range of compound concentrations (0, 10, 30, 100, and 300 μM and 1, 3, and 10 mM). IC_50_ values were calculated from the dose–response curves plotted using GraphPad Prism 5.

### Solid-Phase Extraction Mass Spectrometry-Based Assays

For assays using solid-phase extraction mass spectrometry (SPE-MS) with a 15-mer ssRNA oligonucleotide substrate [AUUGUGG(m^6^A)CUGCAGC], a 25 μL of reaction mixture was added 200 μM ascorbic acid; 1 μM 2OG; 0.5 μM (NH_4_)_2_Fe(SO_4_)_2_·6H_2_O; 25 mM Tris, pH 7.5; 0.6 μM 15-mer ssRNA substrate; and 0.1 μM FTO, with a range of compound concentrations (3, 10, 30, 100, and 300 nM, 1, 3, 10, 30, and 100 μM). Reactions were incubated at 25 °C for 10 min before quenching with 10 mM MgCl_2_. A RapidFire RF 365 high-throughput sampling robot (Agilent) attached to an iFunnel Agilent 6550 accurate mass quadrupole time-of-flight (Q-TOF) mass spectrometer operated in the positive ionization mode was used for sample analyses. The samples were aspirated under vacuum for 0.6 s and loaded onto a C-8 reverse phase solid-phase extraction (SPE) cartridge. The cartridge was washed with 6 mM octylammonium acetate (prepared by mixing 0.1 M of octylamine and 0.1 M of acetic acid with 100 mL diethyl ether on ice; this solution was cooled to −40 °C resulting in OAA crystals, which were collected and washed with cold cyclohexane.) in the LC-MS grade water treated with diethyl pyrocarbonate (5.5 s, 1.5 mL/min). The hydroxylated product [AUUGUGG(hm^6^A)CUGCAGC] and the substrate [AUUGUGG(m^6^A)CUGCAGC] were eluted from the SPE cartridge with 6 mM OAA 80/20_v/v_ acetonitrile/water into a mass spectrometer (5.5 s, 1.5 mL/min). The SPE cartridge re-equilibrated with 6 mM OAA in water (0.5 s, 1.5 mL/min). Mass spectrometer parameters: capillary voltage (4000 V), nozzle voltage (0 V), fragmentor voltage (250 V), gas temperature (280 °C), gas flow (13 L/min), nebulizer (40 psi), sheath gas temperature (350 °C), and sheath gas flow (12 L/min). Signal intensities were quantified as the total ion count and analyzed using RapidFire Integrator software (Agilent). Percentage hydroxylation was calculated by comparing the ion intensities of *m*/*z* peaks corresponding to [M – H]^3–^ to [M + OH]^3–^: % hydroxylation = 100 × (product)/(substrate + product). IC_50_ values were calculated from the dose–response curves plotted using GraphPad Prism 5.

### PHD2 and FIH Inhibition Assays

PHD2, FIH, and KDM inhibition assays were performed using a solid-phase extraction–mass spectrometry-based methods as described.^34^ KDM inhibition assays were performed using an AlphaScreen-based assay as described.^35^

### NMR-Based Binding Studies

Apo-FTO was made by treatment with EDTA as follows: fractions containing purified FTO at a concentration of >1 mg/mL in 200 mM EDTA and 15 mM ammonium acetate (pH 7.0 to 8.0) was incubated overnight at 4 °C, then concentrated to 2 mL volume using an Amicon Ultra-4 or Amicon Ultra-15 centrifugal filtration units. Carr–Purcell–Meiboom–Gill (CPMG) displacement experiments were carried out using an AVIII 700 Bruker instrument equipped with a TCI inverse cryoprobe using 3 mm diameter MATCH NMR tubes with 160 μL of sample volume at 298 K. The PROJECT-CPMG sequence of Aguilar et al. was applied (90°_*x*_–[τ–180°_*y*_–τ–90°_*y*_–τ–180°_*y*_–τ]_*n*_–acq)^47^ using: total echo time 48 ms (τ = 2 ms, *n* = 6); acquisition time, 2.94 s; relaxation delay, 2 s; and number of scans 200–400.^38^ Water suppression was achieved by presaturation. Samples containing 10 or 20 μM of apo-FTO, 100 μM of Zn(II), 10 or 20 μM of 2OG, and 20–400 μM of the inhibitor buffered in 50 mM Tris-D_11_/HCl (pH 7.5) and 0.02% NaN_3_ (w/v) in 90% H_2_O and 10% D_2_O (v/v) were analyzed, and the 2OG displacement was measured via the reappearance of free 2OG signals.

The percentage of the 2OG displacement was calculated using:
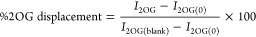
where, *I*_2OG_ = integral of 2OG in the presence of both protein (FTO) and inhibitor; *I*_2OG(0)_ = integral of 2OG in the presence of the protein (FTO) and absence of the inhibitor; *I*_2OG(blank)_ = integral of 2OG in the absence of both protein (FTO) and inhibitor; and the apparent *K*_*D*_ (*K*_D_^app^) of the inhibitors (**13a–d**, **14a–i**) were calculated using reported methods.^38^ The *K*_D_ of 2OG for FTO (1.32 ± 0.5 μM) was used as a reference value.

### RNA Oligonucleotide Synthesis and Purification

RNA synthesis was performed using an Applied Biosystems 394 automated DNA/RNA synthesizer on the 1.0 μmole scale using a standard phosphoramidite cycle of detritylation, coupling, capping, and oxidation. Solid supports were packed into a TWIST column (Glen Research) for synthesis.

For non-modified RNA, 2′-*O*-TC-protected RNA phosphoramidite monomers were used (A-bz, C-ac, G-ib and U, Sigma-Aldrich). Monomers were dissolved at 0.1 M in anhydrous toluene/acetonitrile (1:1 v/v) immediately prior to use. Coupling, capping, and oxidation reagents were identical to those used in standard DNA synthesis except a solution of ethylthiotetrazole (ETT) (0.25 M in acetonitrile, Link Technologies) was used as a coupling reagent (activator). The coupling time for all monomers during RNA synthesis was 3 min. Stepwise coupling efficiencies were determined by the automated trityl cation conductivity measurement and in all cases were >97%. For the cleavage of the RNA from the solid support and deprotection of the bases and sugar, the solid support was exposed to dry ethylenediamine:toluene (1:1 v/v) for 6 h at room temperature, washed with toluene (3 × 1 mL) then acetonitrile (3 × 2 mL), and dried using argon. The crude cleaved RNA was eluted with water.

For *N*^6^-methyladenosine RNA (5-mer and 15-mer)**,** 2′-*O*-TBDMS RNA phosphoramidites (A-tac, C-tac, G-tac, and U, where tac = *tert*-butylphenoxyacetyl; Sigma-Aldrich) were dissolved in anhydrous acetonitrile (0.1 M) immediately prior to use. Coupling, capping, and oxidation reagents were 5-benzylthio-1*H*-tetrazole (0.3 M in acetonitrile; Link Technologies), fast deprotection Cap A (5% *tert*-butyl phenoxyacetyl acetic anhydride in tetra-hydrofuran)/Cap B (16% *N*-methylimidazole in tetrahydrofuran), and iodine (0.1 M in tetrahydrofuran, pyridine, and water), respectively. The coupling time during RNA synthesis was 10 min. Stepwise coupling efficiencies were determined by automated trityl cation conductivity monitoring and in all cases were >97%.

For the cleavage of the RNA from the solid support and deprotection of the bases, the solid support was exposed to concentrated aqueous ammonia:ethanol (3:1 v/v) for 2.5 h at room temperature followed by 2 h at 55 °C in a sealed vial. Ammonia was removed in vacuo. To remove the 2′-TBDMS protecting group, the ammonia-free solution was freeze-dried and re-dissolved in a 1:1 mixture of dry DMSO (300 μL) and triethylaminetrihydrofluoride (300 μL) and heated for 2.5 h at 65 °C. After cooling down to room temperature, sodium acetate (3 M pH 5.2, 50 μL) and butanol (3 mL) were added, and the RNA was stored for 30 min at −80 °C. RNA was then pelleted by centrifugation (12,000*g*, 30 min, 4 °C), the supernatant discarded, and the pellet washed twice with 70% ethanol (750 μL). The pellet was then dried in vacuo and dissolved in water.

Oligonucleotides were purified using a Gilson HPLC system with an ACE C8 reversed-phase column (10 mm × 250 mm, pore size 100 Å, particle size 10 μm) with a gradient of buffer A (0.1 M TEAB, pH 7.5) to buffer B (0.1 M TEAB, pH 7.5 containing 50% v/v acetonitrile) and a flow rate of 4 mL/min. The gradient of acetonitrile in triethylammonium bicarbonate (TEAB) was increased from 0 to 50% buffer B over 30 min. Elution was monitored by ultraviolet absorption at 298 nm. After HPLC purification, the RNA was freeze-dried and then dissolved in water. Purified oligonucleotides were characterized by ESI mass spectrometry using a XEVO G2-QTOF MS instrument. Data were processed using MaxEnt and in all cases confirmed the integrity of the sequences.

### Protein Crystallization

Crystals of the FTOΔ31-**13c** and FTOΔ31-**14a** complexes were grown in hanging drops at 293 K by the vapor diffusion method. For FTOΔ31-**13c** crystallization, 2 μL of the protein solution containing 8 mg/mL FTOΔ31 solution, 1 mM ZnSO_4_, and 1 mM **13c** was mixed with 1 μL of the reservoir solution containing 100 mM trisodium citrate pH 5.6, 8% PEG 3350, and 4% *tert*-butanol to create a crystallization drop. For FTOΔ31-**14a** crystallization, 1 μL of solution containing 8 mg/mL of the FTOΔ31-**14a** complex and 1 mM NADH was mixed with 1 μL of reservoir solution containing 100 mM trisodium citrate pH 5.6, 13.5% PEG 3350, 4% *tert*-butanol to create a crystallization drop. The drops were equilibrated with 200 μL of the reservoir solution. The resultant crystals were cryoprotected by soaking crystals in the reservoir solution diluted with 20% (v/v) glycerol before being flash cooled in liquid nitrogen.

Crystals of the AlkBΔN11-**14a** complex were grown using the sitting drop method at 293 K. The volume ratio of the protein to reservoir was 1:2. 1 μL of the protein solution containing 20 mg/mL AlkBΔN11, 2 mM MnCl_2_, and 2mM of **14a** were mixed with 2 μL of the reservoir solution containing 0.1 M NaCl, 21% w/v PEG 3350, 0.1 M HEPES to create a crystallization drop. The resultant crystals were cryoprotected by soaking crystals in the reservoir solution diluted with 20% (v/v) glycerol before being flash cooled in liquid nitrogen.

### X-ray Data Collection, Structure Determination, and Refinement

Data were collected at Diamond Light Source beamlines I04 or I24 equipped with a Pilatus 6M-F (FTOΔ31-**13c** and AlkBΔN11-**14a**) or Dectris EIGER2 9M (FTOΔ31-**14a**) detector, respectively. Data for FTOΔ31-**13c** were processed in-house using HKL2000^48^ and data for FTOΔ31-**14a** and AlkBΔN11-**14a** were processed by the beamlines autoprocessing pipeline using the XIA2 strategy.^49^ FTO structures were solved by molecular replacement (MR) using PHASER^50^ with a structure of FTOΔ31-**4** as the search model (PDB ID 4IE5), followed by iterative cycles of map fitting in COOT^53^ and refinement using PHENIX.^51^ The structure of AlkBΔN11-**14a** was solved by MR using PHASER and PDB ID 2FDJ as the search model. Iterative cycles of model building using COOT and refinement with PHENIX were performed until the converging *R*_work_ and *R*_free_ no longer decreased. **14a** in the FTOΔ31-**14a** structure was refined with an occupancy of 0.7. Residual positive difference densities near the active site were present, likely derived from low occupancy citrate and/or glycerol from the crystallization mother liquor and cryoprotectant and could not be modeled with confidence. Data collection and refinement statistics for all structures can be found in Table S1.

### Synthetic Chemistry Methods

Reagents were from Sigma-Aldrich, Alfa Aesar, Cambridge Biotech, Fischer Scientific, or Link Technology, unless otherwise stated. Anhydrous solvents used in reactions were either analytical grade, as obtained commercially (Alfa Aesar), or were freshly distilled. HPLC grade solvents were employed for work-up and chromatography. For the chromatographic purification of phosphoramidites, solvents were dried over P_2_O_5_ prior to use. Reactions involving moisture-sensitive reagents were carried out under an argon atmosphere; glassware was oven-dried and cooled under nitrogen before use. Reagents were used as supplied (analytical or HPLC grade) without prior purification. Anhydrous MgSO_4_ was used as a drying agent. Microwave experiments were carried out using a Biotage Initiator 8 machine. Thin-layer chromatography was performed using aluminum plates coated with 60 F254 silica. Plates were visualized using UV light (254 nm), or 1% (*m*/*v*) aq KMnO_4_ stain. Flash column chromatography was performed using Kieselgel 60 silica in a glass column, or on a Biotage SP4 flash column chromatography platform. Retention factors (*R*_f_) are quoted to a precision of 0.05.

Deuterated solvents were from Sigma and Apollo Scientific Ltd. ^1^H NMR and ^13^C NMR spectra were recorded using Bruker AVIII400, AVIII500, AVIII600, and AVIII700 NMR spectrometers. Fields were locked by external referencing to the relevant residual deuterium resonance. Chemical shifts (δ) are reported in ppm; coupling constants (*J*) are recorded in Hz to the nearest 0.5 Hz; when peak multiplicities are reported, the following abbreviations are used: s = singlet, d = doublet, t = triplet, q = quartet, m = multiplet, br = broadened, dd = doublet of doublets, dt = doublet of triplets, and td = triplet of doublets. Spectra were recorded at room temperature unless otherwise stated.

Low-resolution mass spectra (*m*/*z*) and high-resolution mass spectra (HRMS) were recorded using an LCT Premier XE (Waters, MA, USA) or a microTOF (Bruker, MA, USA).

Melting points were recorded on a Gallenkamp Hot Stage apparatus. Purity of synthesized compounds was ≥95% as determined by analytical reverse-phase LC/MS. IR spectra were recorded using a Bruker Tensor 27 FT-IR spectrometer as thin films. The selected characteristic peaks are reported in cm^–1^.

### General Synthetic Procedures

#### General Procedure A: Synthesis of Protected Glycinate Derivatives (Suzuki Coupling) (Scheme S1)

To an overnight dried microwave vial were added the requisite aryl halide (**8, 9**; 1 equiv), K_3_PO_4_ (3 equiv), and the requisite boronic acid (1.2 equiv) in CH_3_CN/H_2_O (3:1). The vial was evacuated and filled with N_2_ three times. To this degassed solid mixture was added Pd(PPh_3_)_4_ (10 mol %); the vial was again evacuated and filled with N_2_ three times. The vial was then subjected to microwave radiation (Biotage Initiator 8 machine, 135 °C, 3 h), pH was raised with 1 M NaOH. The mixture was diluted with ethyl acetate and washed with 3 × H_2_O. The combined organic extracts were dried over anhydrous MgSO_4_ and concentrated in vacuo. The crude mixture was chromatographed (cyclohexane/ethyl acetate 8:2 to 7:3) to provide the product.

#### General Procedure B: Synthesis of Protected Glycinate Derivatives (Buchwald Coupling) (Scheme S2)

To an overnight-dried microwave vial were added the appropriate aryl halide (**8–10**; 1 equiv), K_3_PO_4_ (3 equiv), BrettPhos (**16**; 0.14 equiv), and Pd precatalyst (**17**; 0.01 equiv). The vial was evacuated and filled with N_2_ three times. To this degassed solid mixture were added appropriate amine (1.2 equiv) and *t*-BuOH (2.5 mL). The vial was again evacuated and filled with N_2_ three times. The vial was then subjected to microwave heating (Biotage Initiator 8 machine, 8 h, 140 °C). The solvent was removed under reduced pressure and the residue was diluted with ethyl acetate. The organic layer was washed with 3 × H_2_O and crystallized from CH_2_Cl_2_. Yields varied from 22-86%.

#### General Procedure C: Benzyl Group Deprotection (Scheme S3)

To a stirred solution of the appropriate compound (**12c**–**d**; 0.1 mmol) in THF (2 mL) was added Pd/C (10 mol %). The reaction mixture was evacuated and filled with N_2_ three times. The reaction mixture was stirred for 24 h under a H_2_ atmosphere. The suspension was filtered through celite pad; the filtrate was concentrated under reduced pressure. The oily residue was washed with methanol to afford the desired acid.

#### General Procedure D: *tert*-Butyl Group Deprotection (Scheme S4)

To a stirred solution of compound (**11a**–**d**, **12a**–**b**, **12e**–**i**, 0.1 mmol) in THF was added excess KOH; the reaction was stirred for 2 days at room temperature. The crude product mixture was concentrated under reduced pressure, then diluted with ethyl acetate and water. The aqueous layer was acidified with 1 M HCl to pH 3. The resulting precipitate was filtered and washed with cold water (5 mL).

##### 6-Bromo-3-hydroxypicolinonitrile (**6**)

To a suspension of 3-hydroxypicolinonitrile (1.20 g, 10 mmol) in H_2_O, Br_2_ (0.6 mL, 11.5 mmol) was added portion wise over 3 days at room temperature. The mixture was washed with 10% sodium thiosulfate (50 mL). The product was crystallized from H_2_O, resulting in a white solid (670 mg, 53%). mp 135 °C. *R*_f_ 0.20; IR (neat): ν/cm^–1^ 3150 (b, ArO–H), 2290 (−CN); ^1^H NMR (400 MHz, CD_3_CN): δ 7.37 (d, *J* = 9.0 Hz, 1H), 7.60 (d, *J* = 9.0 Hz, 1H), 11.56 (s, 1H); ^13^C NMR (125 MHz, CD_3_CN): δ 127.5 (ArC), 128.2 (ArC), 130.4 (ArC), 132.9 (ArC), 142.5 (ArC), 156.6 (**C**N); HRMS (ESI) *m*/*z*: calcd for C_6_H_2_^79^BrN_2_O [M – H]^−^, 196.9356; observed, 196.9350.

##### 6-Bromo-3-hydroxypicolinic Acid (**7**)

To a stirred suspension of 6-bromo-3-hydroxypicolinonitrile (**6**; 2.8 g, 14.1 mmol) in methanol (50 mL), 30% w/v NaOH (44 mL, 51.8 mmol) was added. The reaction mixture was heated at reflux for 12 h. The solvent was removed under reduced pressure and the suspension was acidified with concentrated HCl to pH 2. The resulting precipitate was filtered and washed with cold water (5 mL). Filtration afforded product **7** (3.1 g, 100%) as white needles. mp 122–124 °C. IR (neat): ν/cm^–1^ 3100 (ArO–H), 2850 (COO–H), 1716 (HOC=O); ^1^H NMR (400 MHz, DMSO-*d*_6_): δ 7.05–7.10 (m, 1H), 7.33–7.75 (m, 1H), 11.43 (s, 1H); ^13^C NMR (125 MHz, DMSO-*d*_6_) 131.2 (ArC), 133.7 (ArC), 135.8 (ArC), 137.9 (ArC), 143.3 (ArC), 169.1 (**C**=O); HRMS (ESI) *m*/*z*: calcd for C_6_H_3_^79^BrNO_3_ [M – H]^−^, 215.9302; observed, 215.9298.

##### *tert*-Butyl (6-Bromo-3-hydroxypicolinoyl)glycinate (**8**)

To a mixture of 6-bromo-3-hydroxypicolinic acid (**7**; 0.2 g, 0.92 mmol), diisopropylethylamine (0.5 mL, 2.75 mmol), and propylphosphonic anhydride solution (T3P, 0.70 mL, 1.2 mmol) in ethyl acetate (2 mL) was added glycine *tert*-butyl ester hydrochloride (0.2 g, 1.2 mmol). The reaction was subjected to microwave irradiation at 120 °C for 4 h. The mixture was diluted with ethyl acetate and washed with water (3 × 10 mL) and concentrated in vacuo. The residue was purified by column chromatography (9:1 to 6:4 cyclohexane/ethyl acetate) to give a pale yellow solid (0.13 g, 42%). mp 105–106 °C. *R*_f_ 0.40; IR (neat): ν/cm^–1^ 3349 (NH), 1748 (*t*-BuOC=O), 1675 (NHC=O); ^1^H NMR (500 MHz, CDCl_3_): δ 1.44 (s, 9H), 4.04 (d, *J* = 5.5 Hz, 2H), 7.13 (d, *J* = 9.0 Hz, 1H), 7.40 (d, *J* = 9.0 Hz, 1H), 8.10 (br s, 1H), 11.83 (s, 1H); ^13^C NMR (125 MHz, CDCl_3_): δ 28.0 (C–(**C**H_3_)_3_), 41.5 (**C**H_2_), 82.7 (**C**(CH_3_)_3_), 129.0 (ArC), 129.4 (ArC), 131.3 (ArC), 133.2 (ArC), 157.3 (ArC), 167.7 (**C**=O), 168.0 (**C**=O); HRMS (ESI) *m*/*z*: calcd for C_12_H_15_^79^BrN_2_NaO_4_ [M + Na]^+^, 353.0107; observed, 353.0095.

##### Benzyl-(6-bromo-3-hydroxypicolinoyl)glycinate (**9**)

To a mixture of 6-bromo-3-hydroxypicolinic acid (**7**; 0.20 g, 0.92 mmol), DIPEA (0.5 mL, 2.75 mmol), and T3P (0.7 mL, 1.19 mmol) in ethyl acetate (2 mL) was added glycine *tert*-butyl ester hydrochloride (0.2 g, 1.20 mmol). The reaction was subjected to microwave irradiation (120 °C, 4 h). The mixture was diluted with ethyl acetate and washed with water (3 × 10 mL). The organic extracts were combined, dried over MgSO_4_, and then concentrated in vacuo. Chromatography (cyclohexane/ethyl acetate 9:1 to 6:4) afforded the product (0.13 g, 42%) as a pale yellow solid. mp 102–104 °C. *R*_f_ 0.45; IR (neat): ν/cm^–1^ 3391 (Ar-OH), 3035 (N–H), 2949 (COO–H), 1741 (PhOC=O), 1654 (NHC=O); ^1^H NMR (400 MHz, CDCl_3_): δ 4.19 (d, *J* = 6.0 Hz, 2H), 5.17 (s, 2H), 7.15 (d, *J* = 9.0 Hz, 1H), 7.19 (s, 2H), 7.31 (m, 4H), 8.12 (br s, 1H), 11.72 (s, 1H); ^13^C NMR (101 MHz, CDCl_3_): δ 40.9 (**C**H_2_), 67.5 (**C**H_2_), 128.5 (ArC), 128.7 (ArC), 128.7 (ArC), 129.1 (ArC), 129.5 (ArC), 131.2 (ArC), 133.5 (ArC), 135.0 (ArC), 157.4 (ArC), 168.0 (**C**=O), 168.8 (**C**=O); HRMS (ESI) *m*/*z*: calcd for C_15_H_12_^79^BrN_2_O_4_ [M – H]^−^, 362.9996; observed, 362.9981.

##### Methyl (6-Bromo-3-hydroxypicolinoyl)glycinate (**10**)

To a mixture of 6-bromo-3-hydroxypicolinic acid (**7**; 0.2 g, 0.92 mmol), diisopropylethylamine (0.5 mL, 2.75 mmol), and propylphosphonic anhydride solution (T3P, 0.70 mL, 1.2 mmol) in ethyl acetate (2 mL) was added glycine methyl ester hydrochloride (0.3 g, 1.2 mmol). The reaction mixture was subjected to microwave irradiation at 120 °C for 4 h. The mixture was diluted with ethyl acetate, washed with water (3 × 10 mL), and concentrated in vacuo. The residue was purified by column chromatography (7:3 to 5:5 cyclohexane/ethyl acetate) to give **10** (114 mg, 43%) as white crystals, mp 88–89 °C. *R*_f_ 0.36; IR (neat): ν/cm^–1^ 3355 (O–H, N–H), 1749 (MeOC=O), 1651 (NHC=O); ^1^H NMR (500 MHz, CDCl_3_): δ 3.79 (s, 3H), 4.23 (d, *J* = 6.0 Hz, 2H), 7.28 (d, *J* = 9.0 Hz, 1H), 8.07 (d, *J* = 9.0 Hz, 1H), 8.46 (s, 1H), 11.76 (s, 1H); ^13^C NMR (125 MHz, CDCl_3_): δ 40.7 (**C**H_2_), 52.5 (**C**H_3_), 126.1 (ArC), 128.9 (ArC), 131.0 (ArC), 139.8 (ArC), 157.7 (ArC), 169.0 (**C**=O), 169.6 (**C**=O); HRMS (ESI+) calcd for C_9_H_9_^79^BrN_2_NaO_4_ [M + Na]^+^, 310.9635; observed, 310.9623.

##### *tert*-Butyl-*N*-((3-hydroxy-6-phenylpyridin-2-yl)carbonyl)glycinate (**11a**)

Following general procedure A; **8** (0.20 g, 0.6 mmol) was reacted with phenylboronic acid (88 mg, 0.73 mmol), K_2_CO_3_ (0.25 g, 1.18 mmol) in CH_3_CN/H_2_O (4 mL) with Pd(PPh_3_)_4_ (70 mg, 0.06 mmol). Chromatography afforded compound **11a** (35 mg, 21%) as colorless oil. IR (neat): ν/cm^–1^ 3400 (N–H), 3090 (C–H), 1737 (*t*BuOC=O), 1652 (NHC=O); ^1^H NMR (400 MHz, CDCl_3_): δ 1.56 (s, 9H), 4.19 (d, *J* = 5.5 Hz, 2H), 7.45–7.40 (m, 2H), 7.52–7.47 (m, 2H), 7.84 (d, *J* = 9.0 Hz, 1H), 7.97–7.94 (m, 2H), 8.59 (t, *J* = 5.0 Hz, 1H), 11.95 (s, 1H); ^13^C NMR (101 MHz, CDCl_3_): δ 28.1 (C(**C**H_3_)_3_), 41.6 (**C**H_2_), 82.6 (**C**(CH_3_)_3_), 126.0 (ArC), 126.3 (Ar C), 127.1 (ArC), 128.6 (2ArC), 128.8 (2 ArC), 130.3 (ArC), 138.1 (ArC), 147.7 (ArC), 156.9 (ArC), 160.9 (**C**=O), 168.2 (**C**=O); HRMS (ESI) *m*/*z*: calcd for C_18_H_20_N_2_NaO_4_ [M + Na]^+^, 351.1321; observed, 351.1315.

##### *tert*-Butyl *N*-((6-(biphenyl-2-yl)-3-hydroxypyridin-2-yl)carbonyl)glycinate (**11b**)

Following general procedure A; **8** (0.20 g, 0.60 mmol) was reacted with (1,1′-biphenyl)-2-ylboronic acid (0.10 g, 0.72 mmol), K_2_CO_3_ (0.25 g, 1.18 mmol) in CH_3_CN/H_2_O (4 mL) with Pd(PPh_3_)_4_ (70 mg, 0.0604 mmol). Chromatography afforded compound **11b** (66 mg, 27%) as yellow oil. IR (neat): ν/cm^–1^ 3400 (N–H), 2978 (Ar C–H), 1743 (tBuOC=O), 1653 (NHC=O); ^1^H NMR (400 MHz, CDCl_3_): δ 1.55 (s, 9H), 4.07 (d, *J* = 6.0 Hz, 2H), 7.22–7.12 (m, 3H), 7.38–7.26 (m, 4H), 7.49–7.46 (m, 3H), 7.73–7.69 (m, 1H), 8.18 (t, *J* = 6.0 Hz, 1H), 11.89 (s, 1H); ^13^C NMR (101 MHz, CDCl_3_): δ 28.1 (C(**C**H_3_)_3_), 41.5 (**C**H_2_), 82.5 (**C**(CH_3_)_3_), 126.8 (ArC), 127.7 (ArC), 128.2 (ArC), 128.5 (ArC), 128.8 (ArC), 129.5 (ArC), 130.2 (ArC), 130.2 (ArC), 130.8 (ArC), 130.7 (ArC), 133.8 (ArC), 137.1 (ArC), 140.8 (ArC), 141.6 (ArC), 148.1 (ArC), 156.2 (ArC), 160.1 (**C**=O), 167.6 (ArC), 168.1 (ArC), 168.7 (**C**=O); HRMS (ESI) *m*/*z*: calcd for C_24_H_24_N_2_NaO_4_ [M + Na]^+^, 427.1634; observed, 427.1628.

##### *tert*-Butyl *N*-((3-Hydroxy-6-(naphthalen-1-yl)pyridin-2-yl)carbonyl)glycinate (**11c**)

Following general procedure A; **8** (0.20 g, 0.60 mmol) was reacted with naphthalen-1-ylboronic acid (0.13 g, 0.72 mmol), K_2_CO_3_ (0.25 g, 1.2 mmol) in CH_3_CN/H_2_O (4 mL) with Pd(PPh3)_4_ (70 mg, 0.06 mmol). Chromatography afforded compound **11c** (61 mg, 27%) as yellow oil. IR (neat): ν/cm^–1^ 3400 (N–H), 2978 (C–H), 1740 (tBuOC=O), 1652 (NHC=O); ^1^H NMR (400 MHz, CDCl_3_): δ 1.52 (s, 9H), 4.14 (d, *J* = 6.0 Hz, 2H), 7.49 (d, *J* = 8.5 Hz, 1H), 7.60–7.50 (m, 4H), 7.67 (d, *J* = 8.5 Hz, 1H), 7.96–7.93 (m, 2H), 8.09–8.06 (m, 1H), 8.42 (t, *J* = 6.0 Hz, 1H), 12.03 (s, 1H); ^13^C NMR (101 MHz, CDCl_3_): δ 28.0 (C(**C**H_3_)_3_), 41.5 (**C**H_2_), 82.5 (**C**(CH_3_)_3_), 125.3 (ArC), 125.4 (ArC), 125.9 (ArC), 126.6 (ArC), 126.6, (ArC), 127.4 (ArC), 128.5 (ArC), 128.9 (ArC), 130.4 (ArC), 130.5 (ArC), 131.5 (ArC), 133.9 (ArC), 137.3 (ArC), 149.2 (ArC), 156.7 (ArC), 168.1 (**C**=O), 169.1 (**C**=O); HRMS (ESI) *m*/*z*: calcd for C_22_H_22_N_2_NaO_4_ [M + Na]^+^, 401.1472; observed, 401.1462.

##### *tert*-Butyl *N*-((3-Hydroxy-6-(quinolin-5-yl)pyridin-2-yl)carbonyl)glycinate (**11d**)

Following general procedure A; **8** (0.25 g, 0.76 mmol) was reacted with quinolin-5-ylboronic acid (0.16 g, 0.91 mmol), K_2_CO_3_ (0.31 g, 2.3 mmol) in CH_3_CN/H_2_O (4 mL) with Pd(PPh_3_)_4_ (87 mg, 0.07 mmol). Chromatography afforded compound **11d** (70 mg, 24%) as a white solid. mp 197–200 °C. IR (neat): ν/cm^–1^ 3200 (N–H), 1749 (*t*BuOC=O), 1646 (NHC=O); ^1^H NMR (400 MHz, CDCl_3_): δ 1.46 (s, 9H), 4.12 (d, *J* = 6.0 Hz, 2H), 7.40 (dd, *J* = 8.5, 4.0 Hz, 1H), 7.52–7.56 (m, 1H), 7.64–7.68 (m, 2H), 7.74–7.78 (m, 1H), 8.16 (d, *J* = 8.5 Hz, 1H), 8.51–8.47 (m, 2H), 8.94–8.89 (m, 1H), 12.05 (s, 1H); ^13^C NMR (101 MHz, CDCl_3_): δ 28.0 (C(**C**H_3_)_3_), 41.4 (**C**H_2_), 82.6 (**C**(CH_3_)_3_), 121.5 (ArC), 127.1 (ArC), 127.6 (ArC), 128.8 (ArC), 130.2 (ArC), 130.3 (ArC), 132.9 (ArC), 134.1 (2ArC), 137.35 (ArC), 147.9 (ArC), 148.5 (Ar C), 150.4 (ArC), 157.0 (ArC), 168.1 (**C**=O), 168.9 (**C**=O); HRMS (ESI) *m*/*z*: calcd for C_21_H_22_N_3_O_4_ [M + H]^+^, 380.1605; observed, 380.1603.

##### *tert*-Butyl *N*-((3-Hydroxy-6-phenylpyridin-2-yl)carbonyl)glycinate (**13a**)

**11a** (200 mg, 0.6 mmol) was reacted according to general procedure D; concentration in vacuo afforded compound **13a** (50 mg, 30%) as a white solid. mp 141–145 °C. IR (neat): ν/cm^–1^ 3400 (O–H), 3200 (N–H), 3070 (C–H), 1708 (HOC=O), 1652 (NHC=O); ^1^H NMR (400 MHz, DMSO-*d*_6_): δ 4.07 (d, *J* = 6.0 Hz, 2H), 7.46–7.41 (m, 1H), 7.59–7.54 (m, 3H), 8.18 (d, *J* = 9.0 Hz, 1H), 8.25–8.23 (m, 2H), 9.48 (t, *J* = 6.0 Hz, 1H), 12.38 (s, 1H); ^13^C NMR (101 MHz, DMSO-*d*_6_): δ 41.2 (**C**H_2_), 126.3 (ArC), 126.7 (ArC), 127.5 (ArC), 127.8 (ArC), 129.1 (ArC), 129.2 (ArC), 130.5 (ArC), 134.5 (ArC), 137.7 (ArC), 147.0 (ArC), 156.9 (ArC), 169.5 (**C**=O), 171.1 (**C**=O); HRMS (ESI) *m*/*z*: calcd for C_14_H_11_N_2_O_4_ [M – H]^−^, 271.0719; observed, 271.0719.

##### *N*-((6-(Biphenyl-2-yl)-3-hydroxypyridin-2-yl)carbonyl)glycine (**13b**)

Following general procedure D; concentration in vacuo afforded compound **13b** (16 mg, 8%) as off white oil. IR (neat): ν/cm^–1^ 3402 (N–H), 2913 (Ar C–H), 1750 (HOC=O), 1657 (HNC=O); ^1^H NMR (400 MHz, DMSO-*d*_6_): δ 4.09 (s, 2H), 7.09–7.15 (m, 4H), 7.24–7.30 (m, 3H), 7.40–7.43 (m, 1H), 7.45–7.49 (m, 2H), 7.71 (dd, *J* = 6.0, 3.0 Hz, 1H); ^13^C NMR (101 MHz, DMSO-*d*_6_): δ 39.9 (**C**H_2_), 125.3 (ArC), 126.4 (ArC), 127.3 (ArC), 127.9 (ArC), 128.2 (ArC), 128.6 (ArC), 129.2 (ArC), 129.9 (ArC), 130.0 (ArC), 130.2 (ArC), 131.7 (ArC), 132.4 (ArC), 138.0 (ArC), 140.9 (ArC), 141.8 (ArC), 149.5 (ArC), 155.9 (ArC), 169.2 (**C**=O), 171.1 (**C**=O); HRMS (ESI) *m*/*z*: calcd for C_20_H_15_N_2_O_4_ [M – H]^−^, 347.1037; observed, 347.1023.

##### *N*-((3-Hydroxy-6-(naphthalen-1-yl)pyridin-2-yl)carbonyl)glycine (**13c**)

**11c** (200 mg, 0.6 mmol) was reacted according to general procedure D; concentration in vacuo afforded compound **13c** (67 mg, 34%) as a white solid. mp 179–182 °C. IR (neat): ν/cm^–1^ 3361 (O–H), 3200 (N–H), 1749 (HOC=O), 1658 (NHC=O); ^1^H NMR (400 MHz, DMSO-*d*_6_): δ 4.02 (d, *J* = 6.0 Hz, 2H), 7.67–7.54 (m, 5H), 7.80 (d, *J* = 8.5 Hz, 1H), 7.96–7.94 (m, 1H), 8.04 (dd, *J* = 8.0, 1.0 Hz, 2H), 9.17 (t, *J* = 6.0 Hz, 1H), 12.44 (s, 1H); ^13^C NMR (101 MHz, DMSO-*d*_6_): δ 41.2 (**C**H_2_), 121.15 (ArC), 125.6 (ArC), 125.85 (ArC), 126.5 (ArC), 127.1 (ArC), 127.2 (ArC), 127.95 (ArC), 128.8 (ArC), 129.1 (ArC), 130.8 (ArC), 131.1 (ArC), 133.8 (ArC), 137.5 (ArC), 149.2 (ArC), 156.7 (ArC), 169.4 (**C**=O), 170.9 (**C**=O); HRMS (ESI) *m*/*z*: calcd for C_18_H_13_N_2_O_4_ [M – H]^−^, 321.0881; observed, 321.0877.

##### *N*-((3-Hydroxy-6-(quinolin-5-yl)pyridin-2-yl)carbonyl)glycine (**13d**)

Following general procedure D; concentration in vacuo afforded compound **13d** (114 mg, 47%) as off white oil. IR (neat): ν/cm^–1^ 3376 (N–H), 1730 (HOC=O), 1654 (NHC=O); ^1^H NMR (400 MHz, DMSO-*d*_6_): δ 4.00 (d, *J* = 6.0 Hz, 2H), 7.54 (dd, *J* = 8.5, 4.0 Hz, 1H), 7.63 (dd, *J* = 8.5, 2.0 Hz, 1H), 7.78 (dd, *J* = 7.0, 1.0 Hz, 1H), 7.90–7.84 (m, 2H), 8.13–8.11 (m, 1H), 8.43 (ddd, *J* = 8.5, 1.5, 1.0 Hz, 1H), 8.95 (dd, *J* = 4.0, 1.5 Hz, 1H), 9.20 (t, *J* = 6.0 Hz, 1H), 12.45 (s, 1H); ^13^C NMR (101 MHz, DMSO-*d*_6_): δ 41.9 (**C**H_2_), 122.5 (ArC), 126.3 (ArC), 127.5 (ArC), 128.2 (ArC), 130.1 (ArC), 130.8 (ArC), 131.2 (ArC), 134.3 (ArC), 137.8 (ArC), 148.0 (ArC), 148.3 (ArC), 151.0 (ArC), 156.9 (ArC), 169.40 (ArC), 171.0 (**C**=O), 172.6 (**C**=O); HRMS (ESI) *m*/*z*: calcd for C_17_H_12_N_3_O_4_ [M – H]^−^, 322.0833; observed, 322.0828.

##### *tert*-Butyl (3-Hydroxy-6-((4-nitrophenyl)amino)picolinoyl)glycinate (**12a**)

The desired product was prepared according to general procedure B; the use of aryl derivative **8** (40 mg, 0.12 mmol) and 4-nitroaniline (18 mg, 0.13 mmol) afforded **12a** (40 mg, 86%) as a yellow solid. mp 196–200 °C. IR (neat): ν/cm^–1^ 3400 (N–H), 3367 (ArO-H), 1741 (*t*-BuC=O), 1645 (NHC=O), 1502, 1319 (N–O); ^1^H NMR (400 MHz, CDCl_3_): δ 1.56 (s, 9H), 4.15 (d, *J* = 5.0 Hz, 2H), 6.82 (br s, 1H), 7.07 (d, *J* = 9.0 Hz, 1H), 7.36 (d, *J* = 9.0 Hz, 1H), 7.45 (d, *J* = 1.0 Hz, 2H), 8.18 (br s, 1H), 8.24 (d, *J* = 1.0 Hz, 2H), 11.60 (s, 1H); ^13^C NMR (101 MHz, CDCl_3_): δ 28.1 (C(**C**H_3_)_3_), 41.6 (**C**H_2_), 83.0 (**C**(CH_3_)_3_), 116.1 (ArC), 118.9 (ArC), 125.8 (ArC), 127.4 (ArC), 129.6 (ArC), 141.2 (ArC), 144.6 (ArC), 146.9 (ArC), 153.5 (ArC), 168.3 (**C**=O), 168.5 (**C**=O); HRMS (ESI) *m*/*z*: calcd for C_14_H_12_N_4_O_6_ [M – H]^−^, 331.0684; observed, 331.0684.

##### *tert*-Butyl (3-Hydroxy-6-((5-nitropyridin-2-yl)amino)picolinoyl)glycinate (**12b**)

The product was prepared according to general procedure B; the use of aryl derivative **8** (40 mg, 0.12 mmol) and 5-nitropyridin-2-amine (18 mg, 0.13 mmol) afforded **12b** (34 mg, 73%) as a yellow solid. mp 196–200 °C. IR (neat): ν/cm^–1^ 3387 (N–H), 3300 (ArO-H), 1742 (*t*-BuC=O), 1647 (NHC=O), 1504, 1382 (N–O); ^1^H NMR (400 MHz, CDCl_3_): δ 1.47 (s, 9H), 4.07 (d, *J* = 5.0 Hz, 2H), 7.32–7.37 (m, 2H), 7.59 (s, 1H), 7.75 (d, *J* = 9.0 Hz, 1H), 8.10–8.16 (m, 1H), 8.33 (s, 1H), 9.07 (t, *J* = 5.0 Hz, 1H), 11.56 (s, 1H); ^13^C NMR (101 MHz, CDCl_3_): δ 28.1 (C(**C**H_3_)_3_), 41.6 (**C**H_2_), 68.3 (**C**(CH_3_)_3_), 109.4 (ArC), 120.2 (ArC), 122.3 (ArC), 129.4 (ArC), 133.5 (ArC), 138.1 (ArC), 143.1 (ArC), 145.9 (ArC), 154.2 (ArC), 157.1 (ArC), 168.2 (**C**=O), 168.7 (**C**=O); HRMS (ESI) *m*/*z*: calcd for C_17_H_18_N_6_O_8_ [M – H]^−^, 388.1262; observed, 388.1266.

##### Benzyl (3-Hydroxy-6-((4-nitrophenyl)amino)picolinoyl)glycinate (**12c**)

The desired product was prepared according to general procedure B; the use of aryl derivative **9** (40 mg, 0.11 mmol) and 4-nitroaniline (18 mg, 0.13 mmol) afforded **12c** (35 mg, 76%) as an orange solid. mp 194–196 °C. IR (neat): ν/cm^–1^ 3340 (N–H), 3289 (ArO-H), 1732 (*t*-BuC=O), 1633 (NHC=O), 1469, 1319 (NO_2_); ^1^H NMR (400 MHz, DMSO-*d*_6_): δ 4.28 (d, *J* = 6.0 Hz, 2H), 5.20 (s, 2H), 7.32–7.43 (m, 5H), 7.46 (d, *J* = 9.0 Hz, 1H), 7.58 (d, *J* = 9.0 Hz, 1H), 7.99 (d, *J* = 1.0 Hz, 2H), 8.17 (d, *J* = 1.0 Hz, 2H), 8.75 (t, *J* = 6.0 Hz, 1H), 10.99 (s, 1H), 11.99 (s, 1H); ^13^C NMR (101 MHz, DMSO-*d*_6_) 49.0 (**C**H_2_), 66.6 (**C**H_2_), 116.5 (ArC), 126.0 (ArC), 126.4 (ArC), 127.4 (ArC), 128.5 (ArC), 128.9 (ArC), 136.2 (ArC), 139.4 (ArC), 147.0 (ArC), 148.7 (ArC), 149.5 (ArC), 153.0 (ArC), 166.4 (**C**=O), 168.9 (**C**=O); HRMS (ESI) *m*/*z*: calcd for C_21_H_16_N_4_O_6_ [M – H]^−^, 421.1148; observed, 421.1163.

##### Benzyl (3-Hydroxy-6-((5-nitropyridin-2-yl)amino)picolinoyl)glycinate (**12d**)

The desired product was prepared according to general procedure B; the use of aryl derivative **9** (40 mg, 0.11 mmol) and amine 5-nitropyridin-2-amine (18 mg, 0.13 mmol) afforded **12d** (30 mg, 65%) as an orange solid. mp 190–195 °C. IR (neat): ν/cm^–1^ 3352 (N–H), 3340 (Ar–OH), 1740 (MeOC=O), 1644 (BzOC=O), 1605 (NHC=O), 1542, 1324 (N–O); ^1^H NMR (400 MHz, CDCl_3_): δ 4.22 (d, *J* = 6.0 Hz, 2H), 5.21 (s, 2H), 7.25–7.43 (m, 7H), 7.57 (br s, 1H), 7.69–7.78 (m, 1H), 8.11 (br s, 1H), 8.33 (d, *J* = 10.0 Hz, 1H), 9.00–9.21 (m, 1H), 11.47 (s, 1H); ^13^C NMR (101 MHz, CDCl_3_): δ 40.5 (**C**H_2_), 67.6 (**C**H_2_), 109.4 (ArC), 116.5 (ArC), 118.5 (ArC), 120.25 (ArC), 122.8 (ArC), 129.4 (ArC), 133.5 (ArC), 138.1 (ArC), 143.1 (ArC), 145.9 (ArC), 154.2 (ArC), 157.1 (ArC), 168.2 (**C**=O), 168.7 (**C**=O); HRMS (ESI) *m*/*z*: calcd for C_20_H_16_N_5_O_6_ [M – H]^−^, 422.1106; observed, 422.1110.

##### Methyl-4-((5-hydroxy-6-((2-methoxy-2-oxoethyl)carbamoyl)pyridin-2-yl)amino)benzoate (**12f**)

The desired product was prepared according to general procedure B; the use of aryl derivative **10** (40 mg, 0.14 mmol) and methyl 4-aminobenzoate (26 mg, 0.17 mmol) afforded **12f** (32 mg, 64%) as a yellow solid. mp 170–175 °C. ^1^H NMR (500 MHz, DMSO-*d*_6_): δ 3.71 (s, 3H), 3.81 (s, 3H), 4.20 (d, *J* = 6.0 Hz, 2H), 7.17 (d, *J* = 9.0 Hz, 1H), 7.42 (d, *J* = 9.0 Hz, 1H), 7.69–7.74 (m, 2H), 7.88–7.93 (m, 2H), 8.58 (t, *J* = 6.0 Hz, 1H), 9.55 (s, 1H), 11.85 (s, 1H); ^13^C NMR (126 MHz, DMSO-*d*_6_): δ 41.4 (**C**H_2_), 52.1 (**C**H_3_), 52.5 (**C**H_3_), 113.1 (ArC), 116.5 (ArC), 120.8 (ArC), 120.8 (ArC), 126.3 (ArC), 129.8 (ArC), 131.2 (ArC), 131.6 (ArC), 146.5 (ArC), 147.3 (ArC), 152.5 (ArC), 166.6 (**C**=O), 169.6 (**C**=O), 170.3 (**C**=O); HRMS (ESI) *m*/*z*: calcd for C_17_H_18_N_3_O_6_ [M + H]^+^, 360.1190; observed, 360.1191.

##### Methyl-4-((6-((2-(benzyloxy)-2-oxoethyl)carbamoyl)-5-hydroxypyridin-2-yl)amino)benzoate (**12g**)

The desired product was prepared according to general procedure B; the use of aryl derivative **9** (40 mg, 0.11 mmol) and methyl 4-aminobenzoate (20 mg, 0.13 mmol) afforded **12g** (15 mg, 32%) as a yellow solid. mp 185–189 °C. IR (neat): ν/cm^–1^ 3408 (N–H), 3352 (Ar-OH), 1740 (MeOC=O), 1690 (BzOC=O), 1651 (NHC=O); ^1^H NMR (400 MHz, CDCl_3_): δ 3.83 (s, 3H), 4.14–4.25 (m, 2H), 5.19 (s, 2H), 6.59 (s, 1H) 6.97 (d, *J* = 9.0 Hz, 1H), 7.20–7.35 (m, 8H), 7.88–7.97 (m, 2H), 8.13 (t, *J* = 6.0 Hz, 1H), 11.33 (s, 1H); ^13^C NMR (101 MHz, CDCl_3_): δ 41.0 (**C**H_2_), 51.9 (**C**H_3_), 67.6 (**C**H_2_), 116.5 (ArC), 118.5 (ArC), 122.8 (ArC), 127.0 (ArC), 128.6 (ArC), 128.7 (ArC), 128.7 (ArC), 129.4 (ArC), 131.3 (ArC), 135.0 (ArC), 145.2 (ArC), 145.6 (ArC), 152.9 (ArC), 166.9 (**C**=O), 168.8 (**C**=O), 169.3 (**C**=O); HRMS (ESI) *m*/*z*: calcd for C_23_H_21_N_3_O_6_ [M – H]^−^, 434.1357; observed, 434.1359.

##### Benzyl (3-Hydroxy-6-((4-(methylcarbamoyl)phenyl)amino)picolinoyl)glycinate (**12h**)

The desired product was prepared according to general procedure B; the use of aryl derivative **9** (40 mg, 0.11 mmol) and 4-amino-*N*-methylbenzamide (20 mg, 0.13 mmol) afforded **12h** (30 mg, 64%) as a yellow solid. mp 165–167 °C. IR (neat): ν/cm^–1^ 3402 (NH), 3300 (ArOH), 1741 (BzOC=O), 1646 (HNC=O), 1602 (HNC=O); ^1^H NMR (400 MHz, CDCl_3_): δ 2.95 (d, *J* = 6.0 Hz, 3H), 4.20 (d, *J* = 6.0 Hz, 2H), 5.19 (s, 2H), 5.96–6.07 (m, 1H), 6.48 (s, 1H), 6.93 (d, *J* = 9.0 Hz, 1H), 7.20–7.35 (m, 8H), 7.66 (d, *J* = 8.5 Hz, 2H), 8.13 (t, *J* = 6.0 Hz, 1H), 11.30 (s, 1H); ^13^C NMR (101 MHz, CDCl_3_): δ 41.0 (**C**H_2_), 51.9 (**C**H_3_), 67.6 (**C**H_2_), 116.5 (ArC), 118.5 (ArC), 122.8 (ArC), 127.0 (ArC), 128.6 (ArC), 128.7 (ArC), 128.73 (ArC), 129.4 (ArC), 131.3 (ArC), 135.0 (ArC), 145.2 (ArC), 145.6 (ArC), 152.9 (ArC), 166.9 (**C**=O), 168.8 (**C**=O), 169.3 (**C**=O); HRMS (ESI) *m*/*z*: calcd for C_23_H_21_N_4_O_5_ [M – H]^−^, 433.1517; observed, 433.1523.

##### Benzyl (3-Hydroxy-6-((4-(dimethylcarbamoyl)phenyl)amino)picolinoyl)glycinate (**12i**)

The desired product was prepared according to general procedure B; the use of aryl derivative **9** (40 mg, 0.11 mmol) and 4-amino-*N*,*N*-dimethylbenzamide (21 mg, 0.13 mmol) afforded **12i** (32 mg, 65%) as a yellow solid. mp 201–204 °C. IR (neat): ν/cm^–1^ 3323 (b, NH, ArOH), 1750 (BzOC=O), 1632 (HNC=O), 1601 (Me_2_NC=O); ^1^H NMR (400 MHz, CDCl_3_): δ 3.02 (br s, 6H), 4.20 (d, *J* = 6.0 Hz, 2H), 5.18 (s, 2H), 6.38 (s, 1H), 6.94 (d, *J* = 9.0 Hz, 1H), 7.21–7.34 (m, 8H), 7.34–7.39 (m, 2H), 8.16 (t, *J* = 6.0 Hz, 1H), 11.28 (s, 1H); ^13^C NMR (101 MHz, CD_3_COCD_3_-*d*_6_): δ 26.6 (**C**H_3_), 40.8 (**C**H_2_), 66.5 (**C**H_2_), 113.0 (ArC), 116.6 (ArC), 119.5 (ArC), 126.0 (ArC), 128.2 (ArC), 128.5 (ArC), 128.6 (ArC), 129.0 (ArC), 129.2 (ArC), 136.1 (ArC), 143.1 (ArC), 147.5 (ArC), 152.2 (ArC), 169.2 (**C**=O), 169.5 (**C**=O), 170.6 (**C**=O); HRMS (ESI) *m*/*z*: calcd for C_24_H_23_N_4_O_5_ [M – H]^−^, 447.1674; observed, 447.1675.

##### 3-Hydroxy-6-(((4-nitrophenyl)amino)picolinoyl)glycine (**14a**)

The desired product was prepared according to general procedure D; glycinate **12a** (20 mg, 0.05 mmol) afforded **14a** (10 mg, 66%) as a yellow solid. mp > 300 °C. IR(neat): ν/cm^–1^ 3387 (NH), 3386 (ArOH), 2939 (COO–H), 1728 (HOC=O), 1647 (HNC=O), 1577, 1334 (N–O); ^1^H NMR (400 MHz, DMSO-*d*_6_): δ 4.09 (d, *J* = 5.0 Hz, 2H), 7.22 (d, *J* = 9.0 Hz, 1H), 7.47 (d, *J* = 9.0 Hz, 1H), 7.81 (d, *J* = 1.0 Hz, 2H), 8.18 (d, *J* = 1.0 Hz, 2H), 8.83 (br s, 1H), 9.96 (t, *J* = 5.0 Hz, 1H), 11.98 (s, 1H), 12.96 (br s, 1H); ^13^C NMR (101 MHz, DMSO-*d*_6_): δ 28.5 (**C**H_2_), 112.4 (ArC), 121.5 (ArC), 128.6 (ArC), 130.3 (ArC), 133.8 (ArC), 137.5 (ArC), 144.6 (ArC), 158.2 (ArC), 169.0 (ArC), 172.7 (**C**=O), 178.5 (**C**=O); HRMS (ESI) *m*/*z*: calcd for C_16_H_15_N_4_O_5_ [M – H]^−^, 331.0684; observed, 331.0685.

##### 3-Hydroxy-6-(((5-nitropyridin-2-yl)amino)picolinoyl)glycine (**14b**)

The desired product was prepared according to general procedure D; glycinate **12b** (20 mg, 0.05 mmol) afforded **14b** (14 mg, 78%) as a yellow solid. mp > 300 °C. IR (neat): ν/cm^–1^ 3400 (NH), 3300 (ArOH), 2856 (COO–H) 1725 (HOC=O), 1647 (HNC=O), 1577, 1334 (N–O); ^1^H NMR (400 MHz, DMSO-*d*_6_): δ 4.09 (d, *J* = 6.0 Hz, 2H), 7.53 (d, *J* = 9.0 Hz, 1H), 7.77 (d, *J* = 9.0, 1H), 7.84 (d, *J* = 9.0 Hz, 1H), 8.39 (dd, *J* = 9.0, 3.0 Hz, 1H), 8.66 (t, *J* = 6.0 Hz, 1H), 9.09 (d, *J* = 3.0 Hz, 1H), 10.66 (s, 1H), 11.98 (s, 1H); ^13^C NMR (101 MHz, DMSO-*d*_6_): δ 21.5 (**C**H_2_), 110.4 (ArC), 121.9 (ArC), 127.5 (ArC), 129.5 (ArC), 133.8 (ArC), 137.5 (ArC), 144.6 (ArC), 146.0 (ArC), 153.8 (ArC), 158.2 (ArC), 171.0 (**C**=O), 172.4 (**C**=O); HRMS (ESI) *m*/*z*: calcd for C_13_H_10_N_5_O_6_ [M – H]^−^, 332.0636; observed, 332.0636.

##### 6-(((4-Aminophenyl)amino)-3-hydroxypicolinoyl)glycine (**14c**)

The desired product was prepared according to general procedure C; glycinate **12c** (20 mg, 0.05 mmol) afforded **14c** (6 mg, 43%) as a violet solid. mp > 300 °C. IR (neat): ν/cm^–1^ 3298 (b, N–H, ArO–H), 2952 (COO–H), 1720 (HOC=O), 1630 (HNC=O); ^1^H NMR (400 MHz, DMSO-*d*_6_): δ 4.22–4.29 (m, 2H), 5.21 (br s, 2H), 7.30–7.46 (m, 6H), 7.66 (br s, 1H), 8.18 (t, *J* = 6.0 Hz, 1H), 11.70 (br s, 1H); ^13^C NMR (101 MHz, DMSO-*d*_6_): δ 49.0 (**C**H_2_), 116.5 (ArC), 126.0 (ArC), 126.4 (ArC), 128.5 (ArC), 128.6 (ArC), 128.9 (ArC), 129.7 (ArC), 136.2 (ArC), 139.4 (ArC), 153.0 (ArC), 166.4 (**C**=O), 169.8 (**C**=O); HRMS (ESI) *m*/*z*: calcd for C_16_H_14_N_3_O_6_ [M – H]^−^, 302.1015; observed, 302.1008.

##### 6-(((5-Aminopyridin-2-yl)amino)-3-hydroxypicolinoyl)glycine (**14d**)

The desired product was prepared according to general procedure C; glycinate **12d** (20 mg, 0.047 mmol) afforded **14d** (3 mg, 21%) as a violet solid. mp > 300 °C. IR (neat): ν/cm^–1^ 3250 (b, N–H, ArO–H), 3000 (COO–H), 1721 (HOC=O), 1680 (HNC=O); ^1^H NMR (400 MHz, DMSO-*d*_6_): δ 4.16 (d, *J* = 6.0 Hz, 2H), 7.47 (d, *J* = 9.0 Hz, 1H), 7.56–7.62 (m, 1H), 7.84–7.89 (m, 2H), 8.14–8.17 (m, 2H), 8.51 (t, *J* = 6.0 Hz, 1H), 8.98 (s, 1H), 9.14 (s, 1H), 11.49 (br s, 1H), 11.67 (s, 1H); ^13^C NMR (101 MHz, DMSO-*d*_6_): δ 41.6 (**C**H_2_), 109.4 (ArC), 120.2 (ArC), 126.3 (ArC), 128.7 (ArC), 129.6 (ArC), 136.2 (ArC), 143.1 (ArC), 145.9 (ArC), 154.2 (ArC), 157.08 (ArC), 165.8 (**C**=O), 170.2 (**C**=O); HRMS (ESI) *m*/*z*: calcd for C_14_H_13_N_4_O_4_ [M – H]^−^, 301.0942; observed, 301.0942.

##### (3-Hydroxy-6-(naphthalen-2-ylamino)picolinoyl)glycine (**14e**)

The desired product was prepared according to general procedure B; glycinate **8** (40 mg, 0.12 mmol) was reacted with 2-naphthylamine (19 mg, 0.13 mmol) and the crude was reacted according to general procedure D to give **14e** (10 mg, 25%). ^1^H NMR (500 MHz, DMSO-*d*_6_): δ 4.05 (d, *J* = 5.7 Hz, 2H), 7.27 (d, *J* = 9.0 Hz, 1H), 7.37 (d, *J* = 9.0 Hz, 1H), 7.47 (t, *J* = 8.0 Hz, 1H), 7.51–7.56 (m, 2H), 7.59 (d, *J* = 8.0 Hz, 1H), 7.89–7.93 (m, 1H), 7.98 (dd, *J* = 7.5, 1.0 Hz, 1H), 8.24–8.28 (m, 1H), 8.44 (t, *J* = 6.0 Hz, 1H), 8.85 (s, 1H), 11.67 (s, 1H); ^13^C NMR (126 MHz, DMSO-*d*_6_): δ 41.2 (**C**H_2_), 116.1 (ArC), 119.7 (ArC), 122.4 (ArC), 122.8 (ArC), 125.7 (ArC), 126.3 (ArC), 126.7 (ArC), 126.8 (ArC), 128.7 (ArC), 129.5 (ArC), 134.6 (ArC), 135.3 (ArC), 137.2 (ArC), 151.7 (ArC), 169.4 (**C**=O), 171.1 (**C**=O); HRMS (ESI) *m*/*z*: calcd for C_18_H_16_N_3_O_4_ [M + H]^+^, 338.1135; observed, 338.1135.

##### 4-((6-((Carboxymethyl)carbamoyl)-5-hydroxypyridin-2-yl)amino)benzoic Acid (**14f**)

To a solution of the methyl ester (**12f**, 359 mg, 1 mmol) in 1,4-dioxane (5 mL), 1 M lithium hydroxide (50 mg, 2 mmol) was added. The mixture was stirred at room temperature for 24 h until consumption of the starting material and then acidified with acetic acid to pH 3 and diluted with CH_2_Cl_2_. The solution was washed with water, dried over MgSO_4_, and concentrated in vacuo to afford **14f** (100 mg, 30%) as a white solid. mp > 300 °C. ^1^H NMR (500 MHz, DMSO-*d*_6_): δ 4.10 (d, *J* = 5.5 Hz, 2H), 7.32 (d, *J* = 9.0 Hz, 1H), 7.39 (d, *J* = 9.0 Hz, 1H), 7.77 (d, *J* = 9.0 Hz, 2H), 7.87 (d, *J* = 9.0 Hz, 2H), 8.50 (t, *J* = 6.0 Hz, 1H), 9.95 (s, 1H), 11.84 (s, 1H); ^13^C NMR (151 MHz, DMSO-*d*_6_): δ 41.5 (**C**H_2_), 113.0 (ArC), 116.5 (ArC), 120.6 (ArC), 122.0 (ArC), 126.2 (ArC), 129.6 (ArC), 131.2 (ArC), 131.6 (ArC), 146.3 (ArC), 147.6 (ArC), 152.2 (ArC), 167.7 (**C**=O), 169.3 (**C**=O), 171.2 (**C**=O); HRMS (ESI) *m*/*z*: calcd for C_15_H_12_N_3_O_6_ [M – H]^−^, 330.0732; observed, 330.0729.

##### 3-Hydroxy-6-(((4-(methoxycarbonyl)phenyl)amino)picolinoyl)glycine (**14g**)

The desired product was prepared according to general procedure C; glycinate **12g** (20 mg, 0.05 mmol) afforded **14g** (13 mg, 82%) as a white solid. mp 264–267 °C. IR (neat): ν/cm^–1^ 3400 (NH), 3370 (ArOH), 2930 (COO–H), 1710 (MeOC=O), 1682 (HOC=O), 1592 (HNC=O); ^1^H NMR (400 MHz, DMSO-*d*_6_): δ 3.81 (s, 3H), 4.09 (d, *J* = 5.5 Hz, 2H) 7.16 (d, *J* = 9.0 Hz, 1H), 7.41 (d, *J* = 9.0 Hz, 1H), 7.73 (d, *J* = 1.0 Hz, 2H), 7.91 (d, *J* = 1.0 Hz, 2H), 8.49 (t, *J* = 5.5 Hz, 1H), 9.55 (s, 1H), 11.87 (br s, 1H); ^13^C NMR (101 MHz, DMSO-*d*_6_): δ 49.1 (**C**H_2_), 52.0 (**C**H_3_), 116.5 (ArC), 120.5 (ArC), 120.9 (ArC), 126.4 (ArC), 129.8 (ArC), 131.1 (ArC), 146.5 (ArC), 147.2 (ArC), 152.3 (ArC), 166.5 (**C**=O), 169.2 (**C**=O), 171.2 (**C**=O); HRMS (ESI) *m*/*z*: calcd for C_16_H_14_N_3_O_6_ [M – H]^−^, 344.0888; observed, 334.0888.

##### 3-Hydroxy-6-(((4-(methylcarbamoyl)phenyl)amino)picolinoyl)glycine (**14h**)

The desired product was prepared according to general procedure C; glycinate **12h** (30 mg, 0.07 mmol) afforded **14h** (12 mg, 51%) as a yellow solid. mp > 300 °C. IR(neat): ν/cm^–1^ 3402 (NH), 3330 (ArOH), 2946 (COO–H), 1728 (HOC=O), 1645 (HNC=O), 1602 (HNC=O); ^1^H NMR (400 M, DMSO-*d*_6_): δ 2.77 (d, *J* = 4.5 Hz, 3H), 4.08 (d, *J* = 5.5 Hz, 2H), 7.13 (d, *J* = 9.0 Hz, 1H), 7.39 (d, *J* = 9.0 Hz, 1H), 7.65 (d, *J* = 1.0 Hz, 2H), 7.80 (d, *J* = 1.0 Hz, 2H), 8.18–8.21 (m, 2H), 8.52 (t, *J* = 5.5 Hz, 1H), 9.31 (s, 1H), 11.82 (s, 1H); ^13^C NMR (101 MHz, DMSO-*d*_6_): δ 26.6 (**C**H_3_), 49.1 (**C**H_2_), 116.4 (ArC), 120.2 (ArC), 126.3 (ArC), 128.7 (ArC), 129.6 (ArC), 144.6 (ArC), 147.6 (ArC), 152.0 (ArC), 166.8 (ArC), 169.4 (ArC), 171.1 (**C**=O), 180.0 (**C**=O), 208.8 (**C**=O); HRMS (ESI) *m*/*z*: calcd for C_16_H_15_N_4_O_5_ [M – H]^−^, 343.1048; observed, 343.1048.

##### 6-(((4-(Dimethylcarbamoyl)phenyl)amino)-3-hydroxypicolinoyl)glycine (**14i**)

The desired product was prepared according to general procedure C; glycinate **12i** (12 mg, 0.03 mmol) afforded **14i** (3 mg, 30%) as a white solid. mp > 300 °C. IR(neat): ν/cm^–1^ 3449 (NH), 3387 (ArOH), 2863 (COO–H), 1730 (HOC=O), 1647 (HNC=O), 1592 (HNC=O); ^1^H NMR (400 MHz, DMSO-*d*_6_): δ 2.98 (s, 6H), 4.02 (d, *J* = 5.5 Hz, 2H), 7.12 (d, *J* = 9.0 Hz, 1H), 7.37 (d, *J* = 8.0 Hz, 3H), 7.66 (d, *J* = 9.0 Hz, 2H), 8.50 (t, *J* = 5.5 Hz, 1H), 9.27 (s, 1H), 11.82 (br s, 1H); ^13^C NMR (101 MHz, DMSO-*d*_6_): δ 21.5 (**C**H_3_), 49.1 (**C**H_2_), 116.6 (ArC), 112.0 (ArC), 126.3 (ArC), 127.9 (ArC), 129.0 (ArC), 129.6 (ArC), 143.2 (ArC), 147.7 (ArC), 151.8 (ArC), 169.2 (**C**=O), 170.7 (**C**=O), 171.0 (**C**=O); HRMS (ESI) *m*/*z*: calcd for C_14_H_12_N_4_O_6_ [M – H]^−^, 357.1204; observed, 357.1205.

##### (*E*)-4-(2-(4-Benzylnicotinoyl)hydrazineyl)-4-oxobut-2-enoic Acid (**15**)^21^

Compound **15** was synthesized as reported.^21^^1^H NMR (600 MHz, DMSO-*d*_6_): δ 4.21 (s, 2H), 6.69 (d, *J* = 15.5 Hz, 1H), 7.09 (d, *J* = 15.5 Hz, 1H), 7.55–7.16 (m, 7H), 8.63 (d, *J* = 37.3 Hz, 1H), 10.91 (s, 1H),10.96 (s, 1H); ^13^C NMR (151 MHz, DMSO-*d*_6_): δ 37.2 (**C**H_2_), 126.6 (**C**H), 128.6 (2ArC, **C**H), 129.2 (2ArC), 131.4 (2ArC), 134.2 (ArC), 138.9 (ArC), 146.3 (ArC), 151.2 (ArC), 162.3 (**C**=O), 166.1 (2**C**=O); HRMS (ESI) *m*/*z*: calcd for C_17_H_16_N_3_O_4_ [M – H]^−^, 326.1141; observed, 326.1135.

#### Synthesis of PhosphoramiditeProtected *N*^*6*^-Methyladenosine (Scheme S5)

##### 3′,5′-*O*-(Di-*tert*-butyl)silyl-2′-*O*-dimethyl(*tert*-butyl)silylinosine (**20**)^52^

The desired compound was prepared according to a modified version of the reported procedure.^52^ To a stirred suspension of inosine (2.12 g, 8 mmol) in 40 mL of anhydrous DMF at 0 °C, di-*tert*-butylsilylditrifluoromethanesulfonate (3.0 mL, 8.8 mmol) was added dropwise under an N_2_ atmosphere. After consumption of the starting material (30 min, as assessed by TLC), the reaction was quenched immediately with imidazole (2.7 g, 40 mmol) at 0 °C. After 5 min, the reaction was warmed to room temperature. *tert*-Butyldimethylsilyl chloride (1.5 g, 9.6 mmol) was then added portionwise, and the reaction mixture was refluxed at 60 °C for 12 h. The suspension was then cooled to room temperature, water was added, and the precipitate was collected by suction filtration. The filtrate was discarded, and the white precipitate was washed with cold methanol. The methanol layer was evaporated under reduced pressure and the product was crystallized from CH_2_Cl_2_ to give a white solid (4.0 g, 98%). m.p 191–193.4 °C. *R*_f_ 0.45 (3:2 cyclohexane/ethyl acetate); ^1^H NMR (600 MHz, CDCl_3_): δ 0.17 (s, 3H), 0.18 (s, 3H), 0.96 (s, 9H), 1.07 (s, 9H), 1.10 (s, 9H), 4.02–4.09 (m, 1H), 4.25 (td, *J* = 10.0, 5.0 Hz, 1H), 4.38 (dd, *J* = 9.5, 4.5 Hz, 1H), 4.45–4.58 (m, 2H), 5.96 (s, 1H), 7.87 (s, 1H), 8.11 (s, 1H), 12.56 (s, 1H); ^13^C NMR (151 MHz, CDCl_3_): δ −5.0, −4.3, 18.3, 20.4, 22.8, 25.9, 27.0, 27.5, 67.8, 74.8, 75.89, 75.94, 92.3, 125.5, 138.3, 144.7, 148.1, 158.9; HRMS (ESI) *m*/*z*: calcd for C_24_H_43_O_4_N_5_^28^Si_2_ [M + H]^+^, 523.2767; observed, 523.2756.

##### 3′,5′-*O*-Bis(*tert*-butyl)silyl-2′-*O*-(*tert*-butyldimethyl)silyl-*N*^6^-methyladenosine (**21**)

The desired compound was prepared according to a modified version of the reported procedure.^52^ To a stirred solution of 3′,5′-*O*-bis(*tert*-butylsilyl)-2′-*O*-(*tert*-butyldimethylsilyl)inosine (**20**; 663 mg, 1.2 mmol) and BOP (0.64 g, 1.44 mmol) in 20 mL of THF, DBU (0.3 mL, 1.8 mmol) was added dropwise and the mixture was heated at 40 °C. After the consumption of the starting material (40 min, as assessed by TLC), the reaction mixture was cooled to room temperature and methylamine (0.3 mL, 6.0 mmol) was added dropwise and the reaction mixture was stirred overnight. The crude product mixture was concentrated under reduced pressure and diluted with ethyl acetate and was washed with water (3 × 10 mL). The organic layer was dried (anhydrous MgSO_4_) and concentrated under vacuum. The residue was purified by column chromatography (9:1 to 3:2 cyclohexane/ethyl acetate) which resulted in oil (665 mg, 98%). *R*_f_ 0.20 (7:3 cyclohexane/ethyl acetate); ^1^H NMR (400 MHz, CDCl_3_): δ 0.00 (s, 3H) 0.02 (s, 3H) 0.78 (s, 9H) 0.90 (s, 9H) 0.94 (s, 9H) 3.05 (d, *J* = 1.0 Hz, 3H) 3.86–3.90 (m, 1H) 4.02–4.10 (m, 1H) 4.34 (dd, *J* = 9.0, 5.0 Hz, 1 H) 4.38–4.44 (m, 1 H) 4.47 (d, *J* = 4.5 Hz, 1 H) 5.76 (br s, 2 H) 7.62 (s, 1 H) 8.22 (s, 1 H); ^13^C NMR (101 MHz, CDCl_3_): δ −5.0, −4.3, 18.3, 20.4, 22.8, 25.9, 27.1, 27.5, 27.6, 67.9, 74.6, 75.5, 75.8, 92.4, 120.5, 125.0, 138.0, 153.4, 155.5; HRMS (ESI) *m*/*z*: calcd for C_25_H_46_O_4_N_5_^28^Si_2_ [M + H]^+^, 536.3082; observed, 536.3078. Analytical data are consistent with those reported.^52^

##### 2′-*O*-(*tert*-Butyldimethyl)silyl-*N*^6^-methyladenosine (**22**)

The desired compound was prepared according to the reported procedure.^52^ To a stirred solution of 3′,5′-*O*-*b*is(*tert*-butylsilyl)-2′-*O*-(*tert*-butyldimethylsilyl)-*N*^6^-methyladenosine (**21**; 240 mg, 0.45 mmol) in 4 mL of CH_2_Cl_2_ at −15 °C, a cooled solution of (HF)_*x*_·pyridine (0.06 mL, 2.3 mmol) in 365 μL of pyridine was added. The reaction temperature was maintained at 0 °C and stirred for 12 h. The reaction was diluted with CH_2_Cl_2_, then washed first with sat. aq NaHCO_3_ solution and with water (3 × 10 mL). The organic layer was dried (anhydrous MgSO_4_) and concentrated under reduced pressure. The residue was purified by column chromatography (9:1 to 3:2 cyclohexane/ethyl acetate) which resulted in oil (160 mg, 90%). *R*_f_ 0.15 (2:3 hexane/ethyl acetate); ^1^H NMR (400 MHz, CDCl_3_): δ 0.00 (s, 3H), 0.02 (s, 3H), 0.94 (s, 9H), 3.42 (d, *J* = 1.0 Hz, 3H), 3.89 (dd, *J* = 10.5, 9.0 Hz, 1H), 4.01–4.11 (m, 1H), 4.34 (dd, *J* = 9.0, 5.0 Hz, 1H), 4.41 (dd, *J* = 9.0, 5.0 Hz, 1H), 4.47 (d, *J* = 5.0 Hz, 1H), 5.76 (s, 2H), 7.62 (s, 1H), 8.22 (s, 1H); ^13^C NMR (101 MHz, CDCl_3_): δ −5.4, −5.3, 17.9, 25.6, 25.8, 27.5, 63.5, 73.1, 74.4, 87.8, 91.3, 119.7, 140.0, 140.1, 152.9, 155.8; HRMS (ESI) *m*/*z*: calcd for C_17_H_30_O_4_N_5_^28^Si [M + H]^+^, 396.2062; observed, 396.2068. Analytical data are consistent with those reported.^52^

##### 5′-*O*-(4,4′-Dimethoxytrityl)–2′-*O*-dimethyl(*tert*-butyl)silyl-*N*^6^-methyladenosine (**23**)

The desired compound was prepared according to the reported procedure.^52^ To a stirred solution of 2′-*O*-dimethyl(*tert*-butyl)silyl-*N*^6^-methyladenosine (**22**; 2.6 g, 6.6 mmol) in 4 mL of anhydrous pyridine at 0 °C, DMTrCl (2.7 g, 8.0 mmol) was added portionwise at regular intervals for 12 h. The reaction was quenched by the addition of an excess of anhydrous methanol (0.5 mL) at room temperature. After 1 h, the solution was concentrated under vacuum. The crude solid was first dissolved and fractioned between aqueous NaHCO_3_ and ethyl acetate; the organic layer was then washed with water (3 × 10 mL). The organic layer was dried (MgSO_4_) and concentrated under vacuum. The residue was purified by column chromatography (9:1 to 3:2 cyclohexane/ethyl acetate) resulting in green oil (3.9 g, 85%). *R*_f_ 0.45 (2:3 cyclohexane/ethyl acetate); ^1^H NMR (400 MHz, CDCl_3_): δ −0.13 (s, 3H) 0.00 (s, 3H) 0.86 (s, 9H) 2.77 (d, *J* = 4.0 Hz, 1H) 3.17 (s, 3H) 3.36–3.43 (m, 1H) 3.54 (dd, *J* = 10.5, 3.5 Hz, 1H) 3.80 (s, 6 H) 4.27 (d, *J* = 3.5 Hz, 1H) 4.33–4.37 (m, 1H) 5.02 (t, *J* = 5.5 Hz, 1H) 5.85 (d, *J* = 4.5 Hz, 1H) 6.04 (br s, 2H) 6.83 (d, *J* = 9.0 Hz, 4H) 7.18–7.28 (m, 3H) 7.36 (d, *J* = 8.0 Hz, 4H) 7.47 (dd, *J* = 8.5 Hz, 1.5, 2H) 7.98 (s, 1H) 8.35 (s, 1H); ^13^C NMR (101 MHz, CDCl_3_): δ −5.6, −5.5, 18.3, 25.8, 25.9, 55.2, 60.4, 63.0, 73.6, 75.5, 85.0, 87.5, 89.2, 113.4, 120.0, 127.3, 128.1, 128.3, 130.39, 130.45, 135.9, 138.0, 145.0, 153.0, 155.4, 158.89, 158.91; HRMS (ESI) *m*/*z*: calcd for C_38_H_48_O_6_N_5_^28^Si [M + H]^+^, 698.3368; observed, 698.3359. Analytical data are consistent with those reported.^52^

##### 5′-*O*-(4,4′-Dimethoxytrityl)-(3′-*O*-[(2cyanoethyl)(*N*,*N*-diisopropylamino)phosphino]-2′-*O*-dimethyl(*tert*-butyl)silyl-*N*^6^-methyladenosine (**24**)

The desired compound was prepared according to the reported procedure.^52^ To a stirred solution of 5′-*O*-(4,4′-dimethoxytrityl)-2′-*O*-dimethyl(*tert*-butyl)silyl-*N*^6^-methyladenosine (**23**, 500 mg, 0.7 mmol) in anhydrous CH_2_Cl_2_ in an over-dried flask under argon, DIPEA (1.3 mL, 7.2 mmol) was added dropwise and the reaction mixture was allowed to stir at 0 °C for 10 min. (2-Cyanoethyl)-*N,N*-diisopropylchlorophosphoramidite (0.40 mL, 1.8 mmol) was added to the reaction mixture dropwise at 0 °C under an argon atmosphere. The reaction was stirred at 0 °C for 30 min, then gradually (about 30 min) warmed to room temperature. After another 5 h under an inert atmosphere, the reaction mixture was treated with a saturated aq KCl solution, then evaporated by rotary evaporation. The desired product was separated by silica gel column chromatography (1:1:0.01 hexane/ethyl acetate/pyridine) resulting in colorless oil (520 mg, 80%) yield. *R*_f_ 0.40 (1:1:0.01 hexane/ethyl acetate/pyridine); ^1^H NMR (700 MHz, CD_2_Cl_2_) Major peaks are listed. δ −0.15 (s, 3H), −0.01 (s, 3H), 0.82 (s, 9H), 1.10 (s,3H), 1.11 (s, 3H), 1.22 (s, 3H), 1.22 (s, 3H), 1.65 (s, 2H), 2.62–2.74 (m, 2H), 3.19 (s, 3H), 3.36 (dd, *J* = 10.5, 4.5 Hz, 1H), 3.54 (dd, *J* = 10.5, 4.0 Hz, 1H), 3.82 (s, 6H), 3.85–3.93 (m, 1H), 3.95–4.10 (m, 1H), 4.41–4.49 (m, 1H), 5.12 (dd, *J* = 6.1, 4.4 Hz, 1H), 5.33–5.40 (m, 2H), 5.79 (s, 1H), 5.99 (d, *J* = 6.0 Hz, 1H), 6.78–6.90 (m, 4H), 7.23–7.29 (m, 1H), 7.28–7.34 (m, 2H), 7.34–7.40 (m, 4H), 7.47–7.52 (m, 2H), 7.94 (s, 1H), 8.25 (s, 1H); ^13^C NMR (176 MHz, CD_2_Cl_2_) major peaks are listed. δ −5.4, −5.0, 0.8, 17.8, 20.4, 20.44, 21.1, 24.37, 24.4, 25.4, 25.44, 42.9, 43.0, 55.2, 58.8, 58.9, 63.5, 72.8, 72.9, 74.7, 74.7, 83.46, 83.48, 86.5, 88.4, 113.1, 117.8, 125.2, 126.8, 127.8, 128.1, 128.2, 129.0, 130.10, 130.14, 135.7, 139.0, 144.9, 153.0, 155.5, 158.6, 158.7; ^31^P NMR (202 MHz, CD_2_Cl_2_): δ 148.0, 150.8.

## Supporting Information Available

Protein crystallography data collection and refinement statistics, supplementary IC_50_ table, IC_50_ curves, reported FTO inhibitors, view of the active site, titration curves, superimposition of the reported AlkB inhibitor binding modes, synthesis of protected glycinate derivatives, *tert*-butyl group deprotection, benzyl group deprotection, and phosphoramidite protected *N6*-methyladenosine, HPLC chromatograms of the selected compounds, and supplementary references (PDF)
Molecular formula strings of compounds (CSV)

## Accession Codes

PDB ID Codes: Atomic coordinates and structure factors of the crystal structures of FTO-**13c** (PDB ID 4QHO), FTO-**14a** (PDB ID 7E8Z), and AlkB-**14a** (PDB ID 7NRO) have been deposited in the RCSB Protein Data Bank. Authors will release the atomic coordinates upon publication.

## Abbreviations

ALKBH: AlkB homologue
CPMG: Carr–Purcell–Meiboom–Gill
DIPEA: diisopropylethylamine
DMSO: dimethylsulfoxide
ESI: electrospray ionization
ETT: ethylthiotetrazole
FIH: factor inhibiting hypoxia-inducible factor
FTO: fat mass- and obesity-associated protein
HFIP: 1,1,1,3,3,3-hexafluoro-2-propanol
HRMS: high-resolution mass spectra
IPTG: isopropyl β-d-1-thiogalactopyranoside
*K*_D_: dissociation constant
KDM: JmjC histone demethylase
LCMS: liquid chromatography–mass spectrometry
m^6^A: *N*^6^-methyladenosine
m^6^Am: *N*^6^-2′*O*-dimethyladenosine
6mA: *N*^6^-methyldeoxyadenosine
m^3^T: *N*^3^-methylthymidine
m^3^U: *N*^3^-methyluridine
NAOX: nucleic acid oxygenase
NMR: nuclear magnetic resonance
2OG: 2-oxoglutarate
PHD: hypoxia-inducible factor prolyl hydroxylase
Q-TOF: quadrupole time-of-flight
RFMS: Rapidfire mass spectrometry
SPE: solid-phase extraction
ssRNA: single-stranded RNA
TET: ten eleven translocation enzyme
