# Supplementary material for: Structure-Based Design of Selective Fat Mass and Obesity Associated Protein (FTO) Inhibitors
Source: J Med Chem. 2021 Nov 11;64(22):16609–25. doi: 10.1021/acs.jmedchem.1c01204 (PMC8631710; doi:10.1021/acs.jmedchem.1c01204)
Supplement: Supplementary file 1 — jm1c01204_si_001.pdf [file jm1c01204_si_001.pdf]

Supporting Information

Structure Based Design of Selective Fat Mass and Obesity Associated Protein (FTO) Inhibitors

Shifali Shishodia[1,3], Marina Demetriades[1], Dong Zhang[1], Nok Yin Tam[2], Pratheesh Maheswaran[1], Caitlin Clunie-O'Connor[1], Anthony Tumber[1], Ivanhoe K. H. Leung[1,5], Yi Min Ng[2], Thomas M. Leissing[1], Afaf H. El-Sagheer[1,4], Eidarus Salah[1], Tom Brown[1], Wei Shen Aik[2,]*, Michael A. McDonough[1,]*, and Christopher Schofield[1,]*

[1] The Chemistry Research Laboratory, Department of Chemistry and the Ineos Oxford Institute for Antimicrobial Research, University of Oxford, 12 Mansfield Road, Oxford, OX1 3TA, United Kingdom.
[2] Department of Chemistry, Hong Kong Baptist University, Kowloon Tong, Hong Kong SAR, China.
[4] Chemistry Branch Department of Science and Mathematics, Suez University, Suez 43721, Egypt.

Present Address
[3] Department of Biochemistry, Medical College of Wisconsin, Milwaukee, Wisconsin 53226, United States.
[5] School of Chemistry and Bio21 Molecular Science and Biotechnology Institute, The University of Melbourne, Parkville, VIC 3010, Australia.

Corresponding Author Information

*(W.S.A.) E-mail: aikweishen@hkbu.edu.hk

*(M.A.M.) E-mail: michael.mcdonough@chem.ox.ac.uk

*(C.J.S.) E-mail: christopher.schofield@chem.ox.ac.uk

Table of Contents

1 **Supplementary Tables and Figures** S3

Table S1: Protein crystallography data collection and refinement statistics. S3

Table S2: IC_{50} values of tested compounds against FTO, KDM5A, KDM5C, and KDM5D S4

Figure S1: S5

Figure S2: S6

Figure S3 ..... S7

Figure S4 ..... S8

Figure S5 ..... S8

Figure S6 ..... S9

Figure S7 ..... S10

Figure S8 ..... S11

Figure S9 ..... S12

Scheme S1 ..... S13

Scheme S2 ..... S13

Scheme S3 ..... S13

Scheme S4 ..... S13

Scheme S5 ..... S14

HPLC chromatograms of selected compounds (14b and 15). S15

2 **Supplementary References** S16

1 Supplementary Tables and Figures

Table S1: Protein crystallography data collection and refinement statistics.

	FTOΔ31-**13c**	FTOΔ31-**14a**	AlkB-**14a**
PDB ID	4QHO	7E8Z	7NRO
**Data Collection**			
Radiation Source	Synchrotron Diamond Beamline I04	Synchrotron Diamond Beamline I24	Synchrotron Diamond Beamline I24
**Detector**	Pilatus 6M-F	Dectris EIGER2 9M	Pilatus 6M-F
**X-ray Wavelength (Å)**	1.2716	0.9999	0.9686
Resolution Range (Å)⌘	50.00 - 2.32 (2.40 - 2.32)	41.08 - 2.55 (2.64 - 2.55)	33.14 - 1.25 (1.30 - 1.25)
Space Group	*R* 3	*R* 3	*P* 1
Unit Cell Dimensions (*a* Å, *b* Å, *c* Å, α°, β°, γ°)	141.38,141.38,83.95, 90°, 90°, 120°	142.29,142.29,83.90, 90°, 90°, 120°	36.88, 38.75, 40.25, 77.60°, 75.38°, 66.14°
Total Number of Reflections Observed	193273 (11597)	65485 (6521)	167312 (15291)
Number of Unique Reflections	26707 (2697)	20587 (2045)	50271 (4947)
Multiplicity⌘	7.2 (4.3)	3.2 (3.2)	3.3 (3.1)
Completeness (%)⌘	100.0 (100.0)	98.9 (93.3)	92.9 (91.3)
$I/\sigma(I)$⌘	11.5 (2.0)	10.4 (0.6)	20.5 (12.9)
R_{merge} (%)	20.4 (N.A.)	8.7 (71.5)	5.1 (7.1)
CC1/2⌘	N.A.	0.990 (0.663)	0.993 (0.992)
**Refinement**			
R_{work} (%)	17.69	19.54	14.63
R_{free} (%)	20.76	23.70	16.41
RMS Deviation (Bonds/Angle)	0.011 Å / 1.22°	0.006 Å/ 0.78°	0.012 Å / 1.20°
**Ramachandran Outliers (%)**	0.2	0	0
Average *B* Factor (Å^2)	86.97	84.82	18.88
Wilson B Factor (Å^2)	67.42	71.21	13.08
Number of Water Molecules	28	12	288

⌘ Numbers in brackets indicate the outermost shell.

$R_{merge} = \sum_j \sum_h | I_{hj} - \langle I_h \rangle | / \sum_j \sum_h \langle I_h \rangle \times 100$.

$R_{work} = \sum ||Fobs| - |Fcalc|| / |Fobs| \times 100$.

R_{free}, based on 7.45% (FTOΔ31-**13c**), 4.59% (FTOΔ31-**14a**) and 4.83% (AlkBΔN11-**14a**) of the total reflections.

Table S2: IC_{50} values of tested compounds against FTO, KDM5A, KDM5C, and KDM5D

	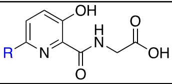	FTO (1.2 μM[a]/100 nM[b])	KDM5A (4.0 nM)	KDM5C (2.0 nM)	KDM5D (2.0 nM)
**13a**	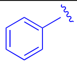	35.2±3.0[a]	87.7[c] (46.1-167.1)	42.0[c] (35.9-49.3)	33.5[c] (26.5-42.3)
**13b**	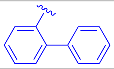	11.1±0.8[a]	50.9[c] (37.9-68.2)	31.0[c] (26.2-36.7)	41.7[c] (31.1-55.8)
**13c**	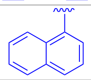	2.3±0.5[a]	>100[c]	>100[c]	>100[c]
**13d**	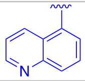	23.2±1.1[a]	32.3[c] (17.8-58.6)	>100[c]	>100[c]
**14a**	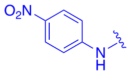	0.08±0.005[b] (1.5±0.5)[a]	81.7[c] (64.2-102)	39.7[c] (30.0-52.6)	27.5[c] (16.8-42.9)
**14b**	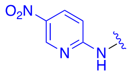	0.33±0.05[b] (12.2±0.8)[a]	>100[c]	>100[c]	>100[c]
**14c**	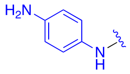	5.1±0.6[b] (60.2±2.7)[a]	53.8[c] (42.9-67.5)	77.7[c] (50.9-118.4)	85.1[c] (54.5-132.8)
**14d**	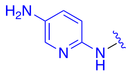	0.8±0.7[b] (6.9±0.6)[a]	>100[c]	>100[c]	>100[c]
**14e**	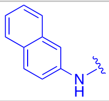	26.0±1.7[b]	*n.d*	*n.d*	*n.d*
**14f**	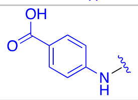	15.2±1.2[b]	*n.d*	*n.d*	*n.d*
**14g**	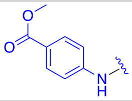	0.9±0.7[b] (5.8±0.4)[a]	>100[c]	>100[c]	>100[c] (59.4-194)
**14h**	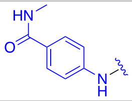	0.43±0.09[b] (6.2±0.4) [a]	63.7[c] (50.2-80.9)	>100[c]	57.8[c] (34.8-96.2)
**14i**	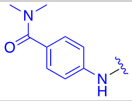	1.4±0.7[b] (54.5±1.9)[a]	>100[c]	>100[c]	>100[c]
**15**	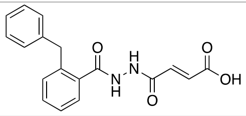	12.5±0.7[a]	>100[c]	>100[c]	>100[c]

IC_{50} values (μM) obtained from the [a]5-mer m^6A RNA LCMS-based demethylation assay, [b] SPE-MS-based assay[1], [c]a histone demethylase luminescence-based AlphaScreen assay[2]. For SPE-MS and AlphaScreen assays the data represent IC_{50} values plus lower and upper 95% confidence limits (in parentheses). Enzyme concentrations employed are stated in parentheses on the first row. The high IC_{50} for **14c** (IC_{50} 5.1 μM) may in part be due its relative lack of solubility at the tested concentrations. Note: n.d., not determined.

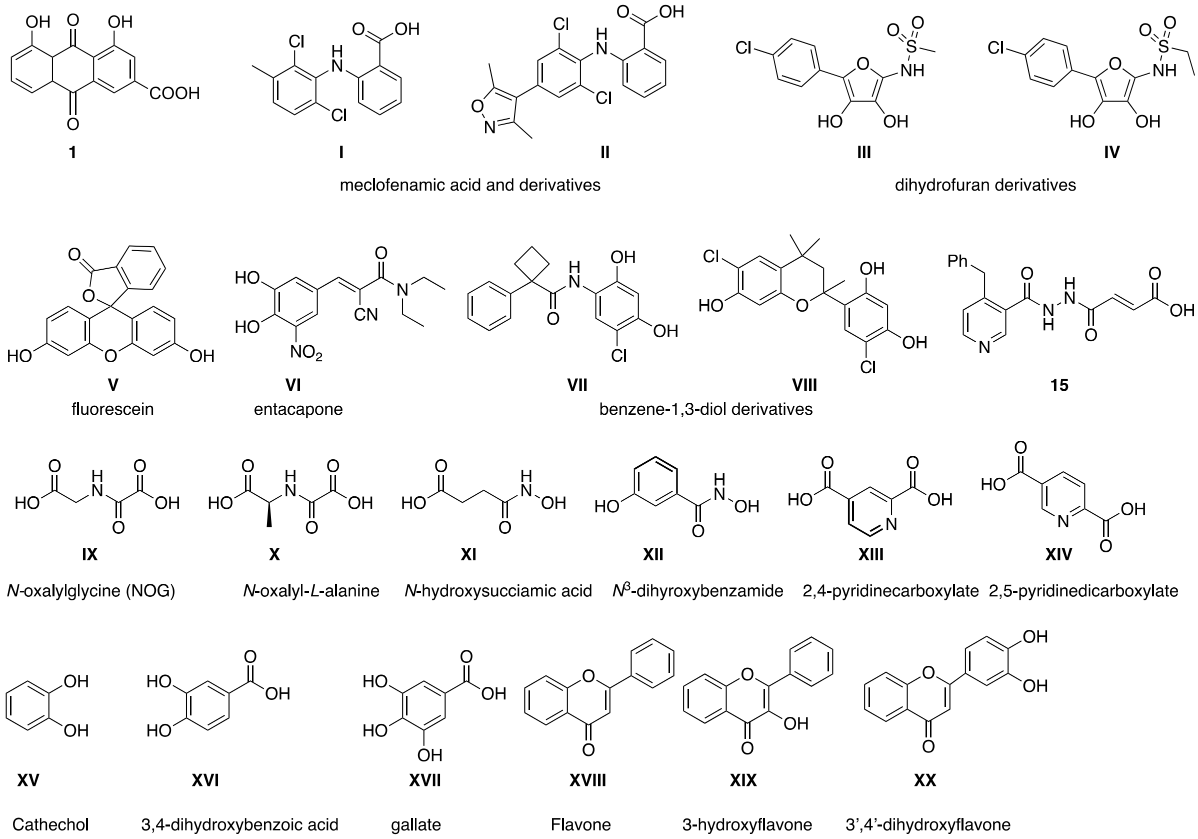

Figure S1: Reported FTO Inhibitors. Rhein(1), meclofenamic acid and derivates (I, II), dihydrofuran derivates (III, IV), fluorescein (V), entacapone (VI), benzene-1,3-diol derivatives (VII, VIII), **15**, generic 2OG oxygenase inhibitors (IX-XX). One of the first reported FTO inhibitors was a tricyclic natural product, rhein (**1**), which was shown to be a substrate competitor[3]. Subsequently, additional FTO inhibitors have been reported, including meclofenamic acid and derivatives (**I**,**II**, Figure S1)[4], dihydoxyfuran derivates (ascorbate analogues, **III**, **IV**, Figure S1)[5], fluorescein (**V**, Figure S1)[6], entacapone derivatives (**VI**, Figure S1)[7], and benzene-1,3-diol derivates (**VII**, **VIII**, Figure S1)[8] and generic 2OG oxygenase inhibitors (**IX**-**XX**, Figure S1; **2**-**4**, Figure 1B)[9–12]. The most potent of the FTO inhibitors reported to date is the fumarate derivative **15**[13]. We have found that **15** is also a potent KDM2A inhibitor (IC_{50} 63 nM).

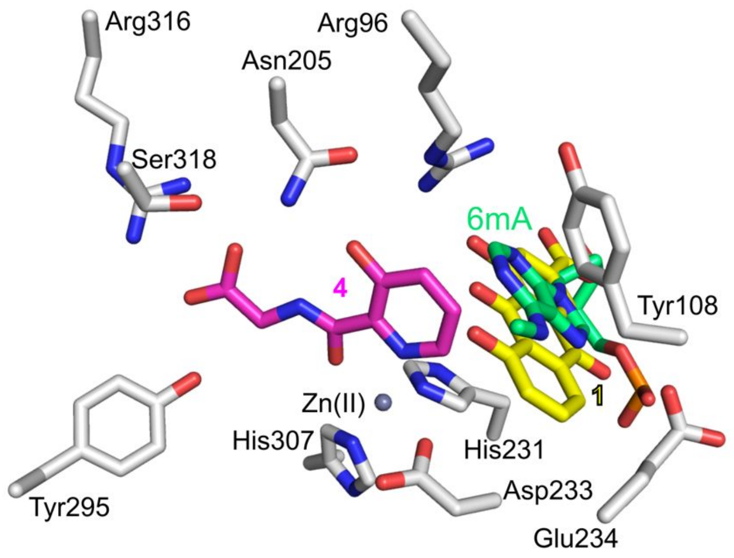

Figure S2: View of the active site from of a crystal structure of FTO∆31 (white sticks) in complex with **4** (magenta sticks) (PDB ID 4IE5) [9]. Structures of FTO∆31 in complex with compound **1** (yellow sticks) (PDB ID 4IE7) [9] and an FTO variant in complex with ssDNA containing *N*[6]-methyldeoxyadenosine (6mA) (PDB ID 5ZMD)[14] are superimposed onto the structure of the FTO∆31-**4** complex (PDB ID 4IE5)[9]. Non-carbon atoms coloured: O (red), N (blue), Zn (grey sphere).

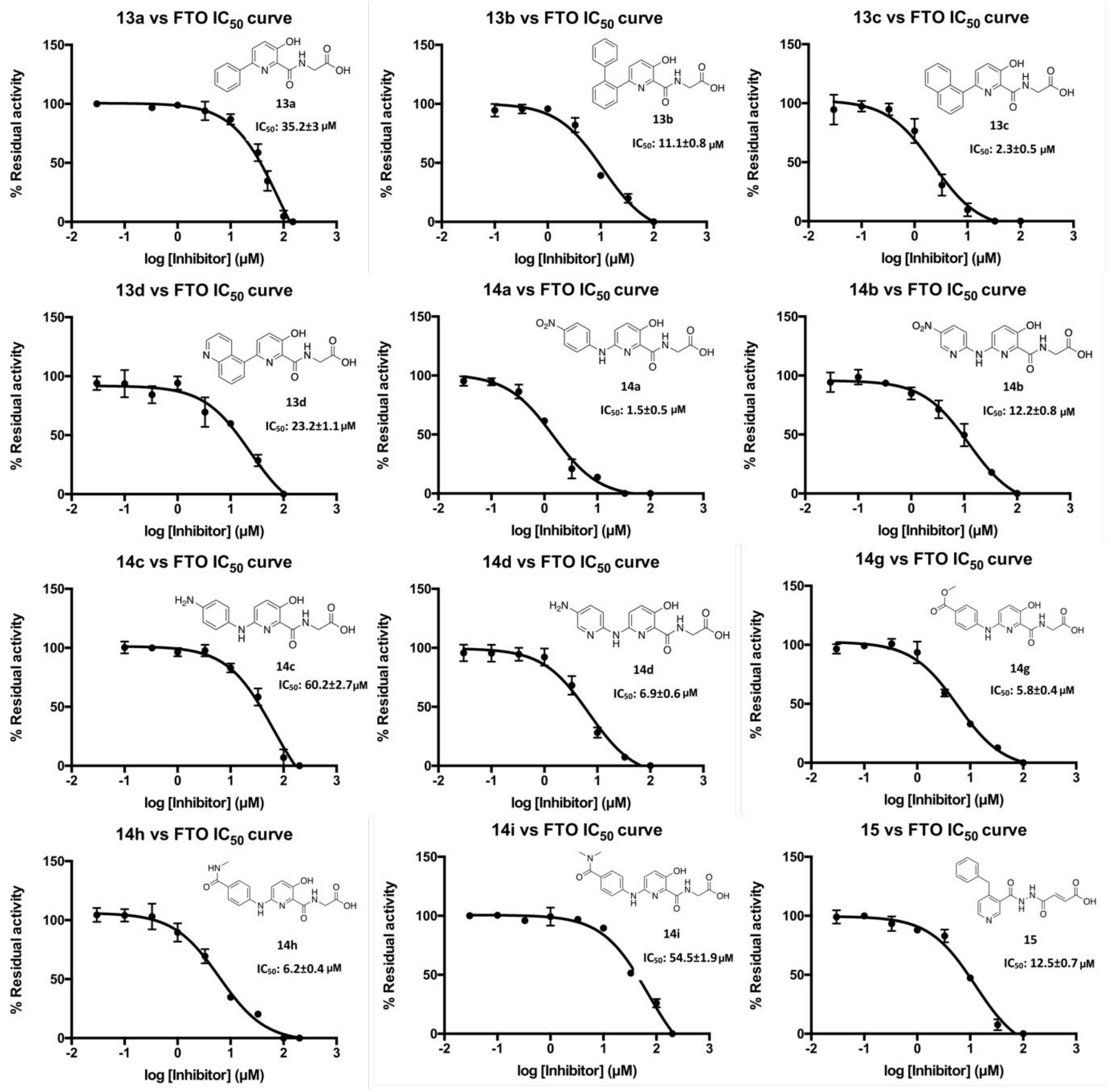

Figure S3: IC_{50} curves from LCMS based demethylation assays using a 5-mer m^6A-containing ssRNA substrate.

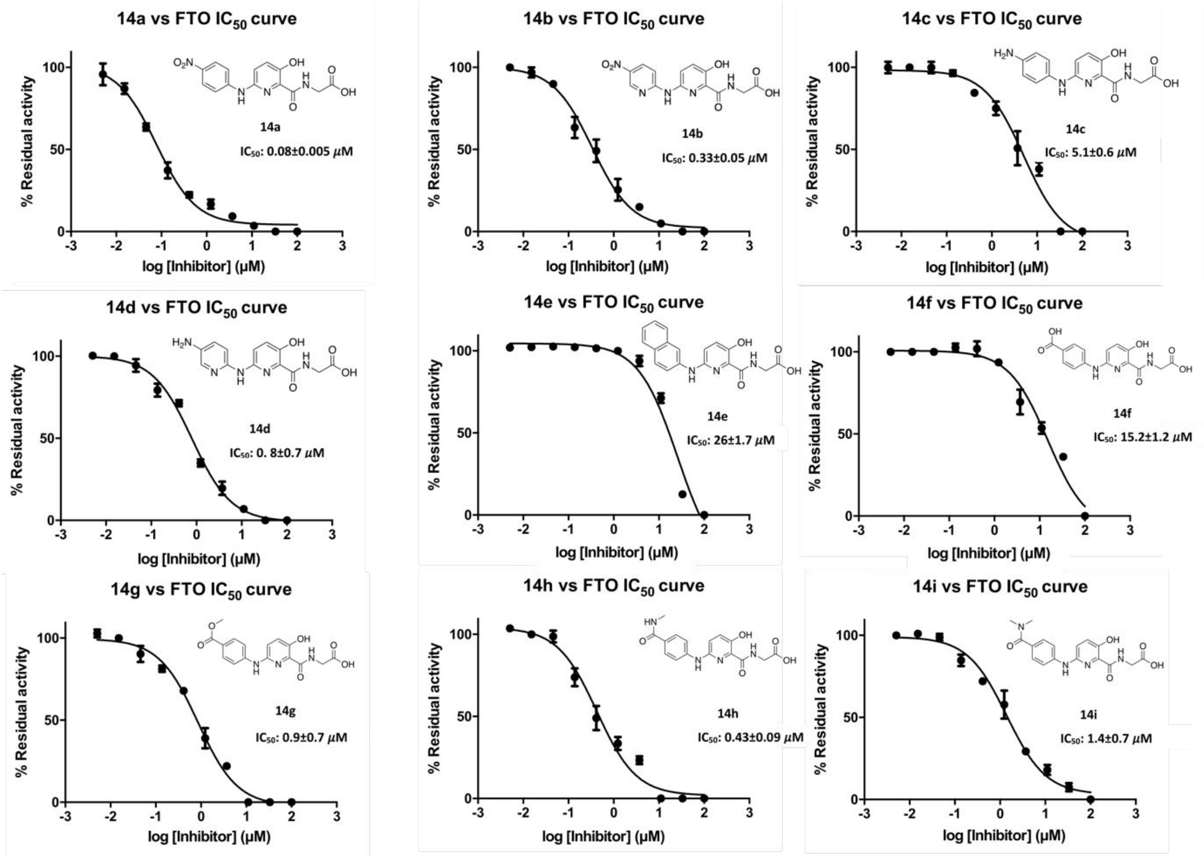

Figure S4: IC_{50} curves from solid phase extraction-mass spectrometry-based hydroxylation assays using a 15-mer m^{6}A-containing ssRNA substrate.

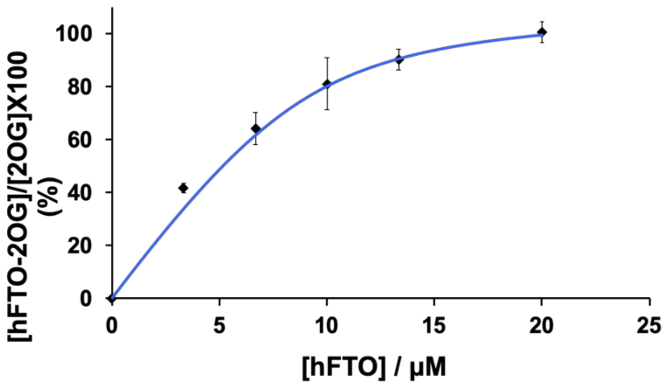

Figure S5: FTO titration curve for K_D^{rep} of 2OG with FTO-Zn(II) complex. K_D^{rep} of 2OG was found to be 1.31±0.47 μM.

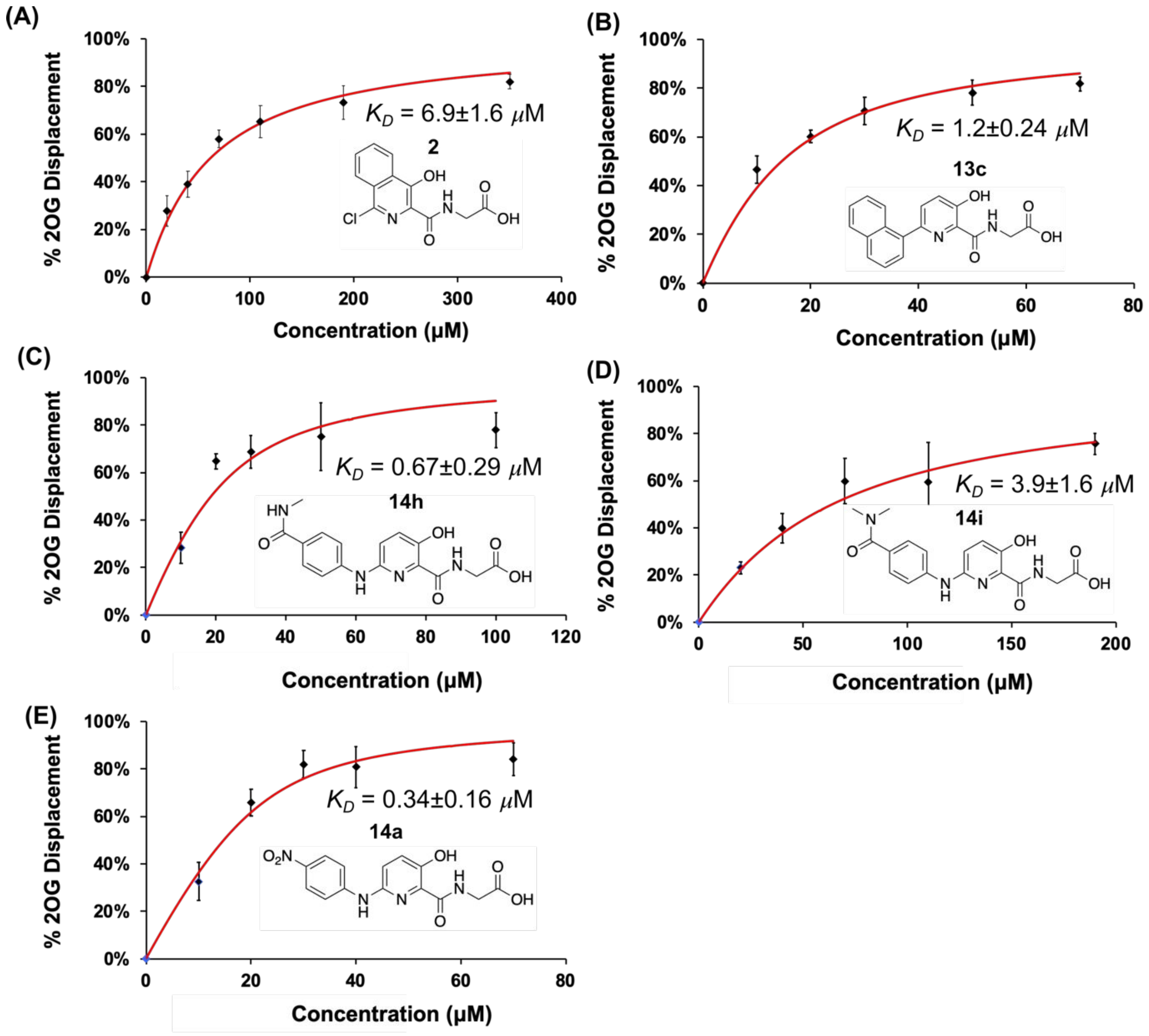

Figure S6: Titration curves for selected FTO inhibitors with 2OG as a competitor. A) **2**; B) **13c**; C) **14h**; D) **14i;** and **E) 14a** were tirated against the FTO.Zn.2OG complex to obtain dissociation constants (K_D) between FTO.Zn and the inhibitors. The calculated K_Ds are 6.9±1.6 μM (**2**), 1.2±0.24 μM (**13c**), 0.67±0.29 μM (**14h**), 3.9±1.6 μM (**14i**), and 0.34±0.16 μM (**14a**).

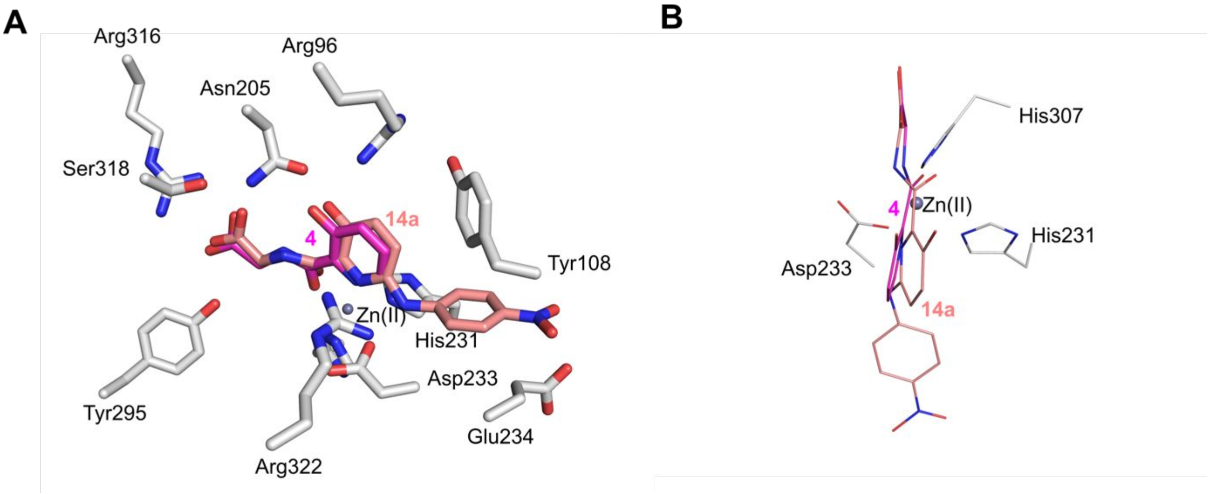

Figure S7: Views of the active site of FTO∆31-**14a** complex (white sticks) (PDB ID 7E8Z) superimposed with views (A, B) of the binding mode of **4** as observed in a FTO∆31-**4** complex structure (PDB ID 4IE5)[9]. **14a**: salmon; non-carbon atoms: O (red), N (blue), Zn (grey sphere).

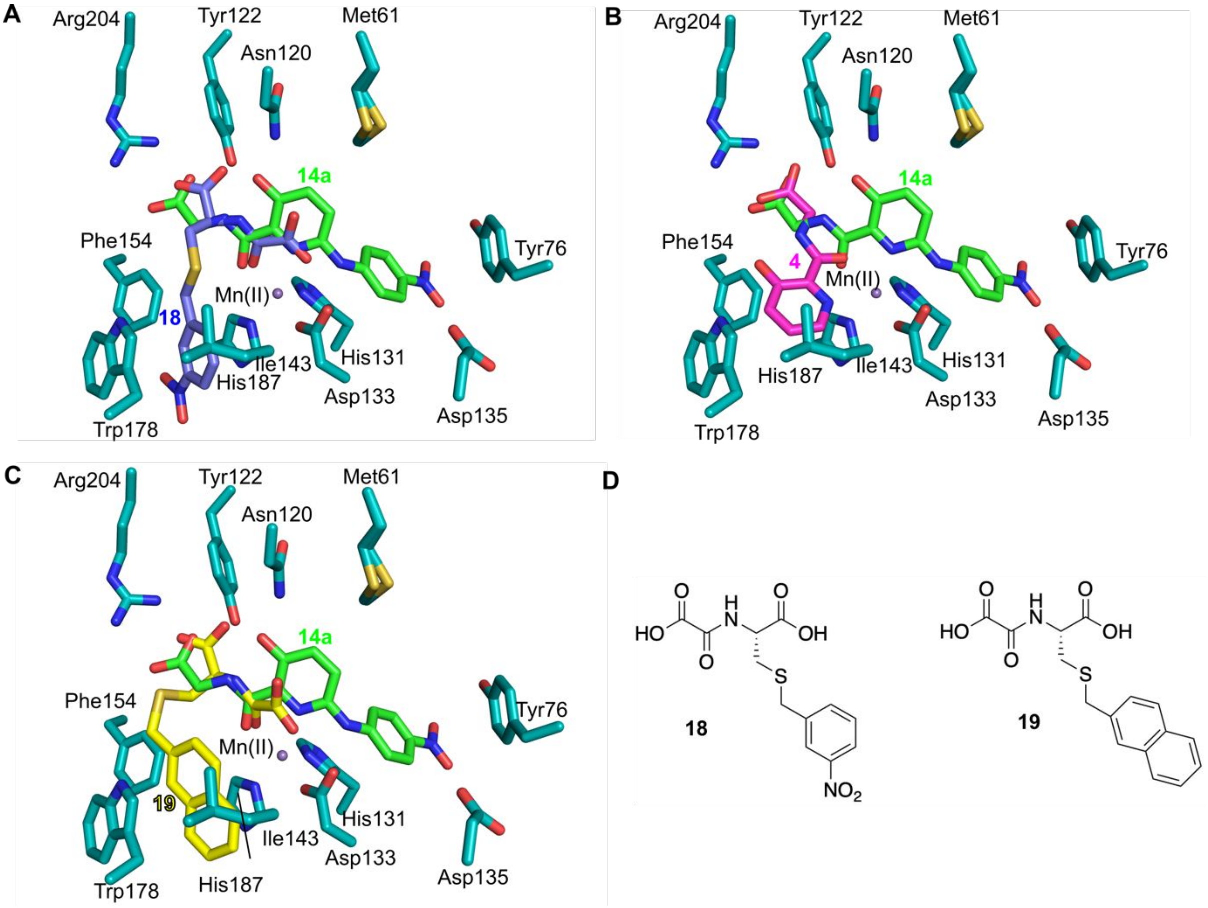

Figure S8: Superimposition of reported AlkB inhibitor binding modes[15] in the active site of a structure of the AlkB△N11-**14a** complex (PDB ID 7NRO). Views of the active site of AlkB△N11-**14a** with the conformations of (A) inhibitor **18** (slate) (PDB ID 3T4H), (B) compound **4** (magenta) (PDB ID 3T3Y), (C) compound **19** (yellow) (PDB ID 3T4V) superimposed on it. Note that the reported AlkB inhibitors **4**, **18** and **19** adopt a 'reverse binding' mode relative to **14a** with respect to metal coordination, leading to their sidechains occupying the hydrophobic pocket by Trp178. (D) Structures of **18** and **19**.

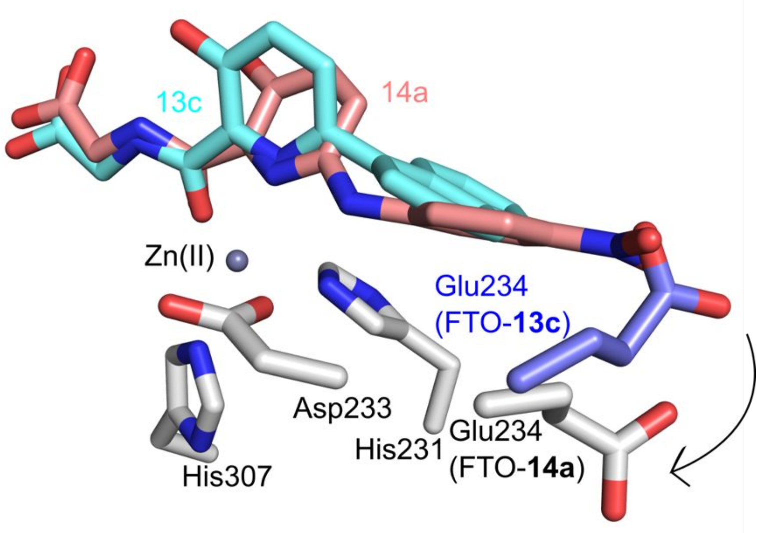

Figure S9: View of the active site of FTO∆31-**14a** complex (PDB ID 7E8Z) superimposed with the conformations of **13c** and Glu234 as observed in a structure of FTO∆31-**13c** (PDB ID 4QHO). The Glu234 sidechain is displaced on binding of **14a** - the nitro group of **14a** occupies the position of the Glu234 sidechain as observed with **13c**.

Supplementary Synthesis Schemes

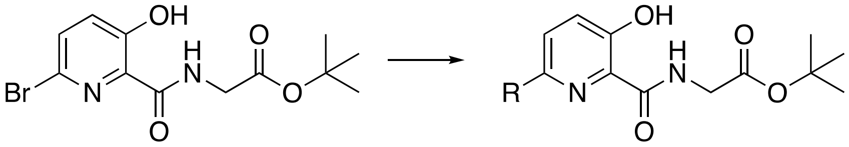

Scheme S1. Synthesis of protected glycinate derivatives (Suzuki coupling).

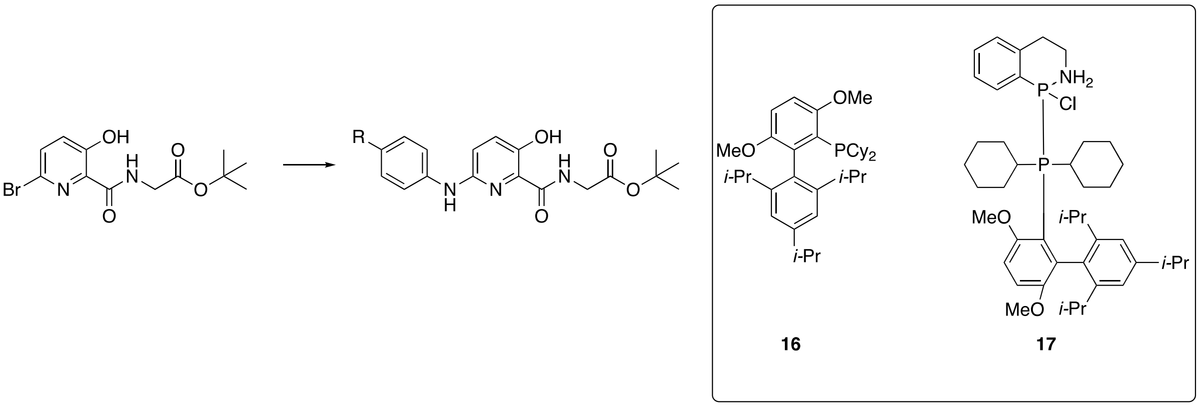

Scheme S2. Synthesis of protected glycinate derivatives (Buchwald coupling).

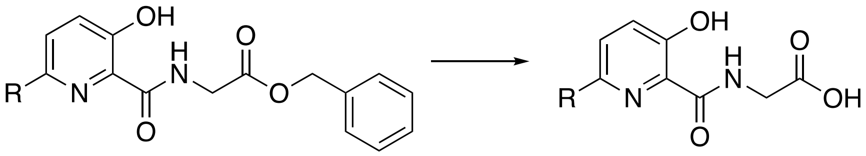

Scheme S3. Benzyl group deprotection

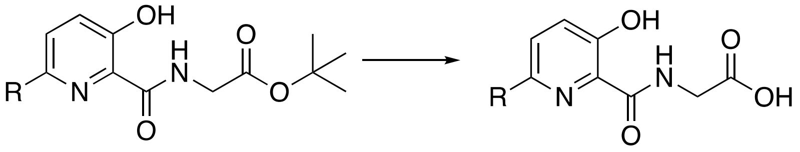

Scheme S4. *tert*-Butyl group deprotection.

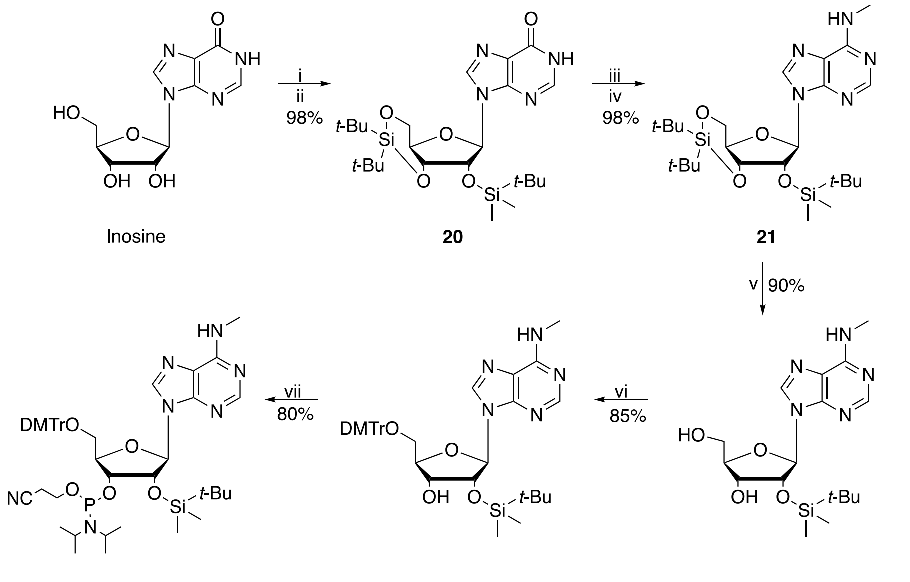

Scheme S5. Synthesis of phosphoramidite protected N^6-methyladenosine. Reaction conditions: i) (t-Bu)$_2$Si(OTf)$_2$, imidazole, DMF, 0 °C, 1 h; ii) TBDMSCl, imidazole, DMF, 60 °C, 12 h; iii) DBU (1.5 equiv.), BOP (1.2 equiv.), THF, 40 °C, 40 min; iv) methylamine (5 eq), rt, 12 h; v) HF.pyridine (3 equiv.), pyridine, 0 °C, 5 h; vi) DMTrCl (1.2 equiv.), pyridine, 0 °C, 12 h; vii) 2-cyanoethyl-*N*,*N*-diisopropylchlorophosphoramidite (2.5 equiv.), diisopropylethylamine (DIPEA, 10 eq), DCM, 0 °C-rt, 6 h.

HPLC chromatograms of selected compounds (**14b** and **15**).

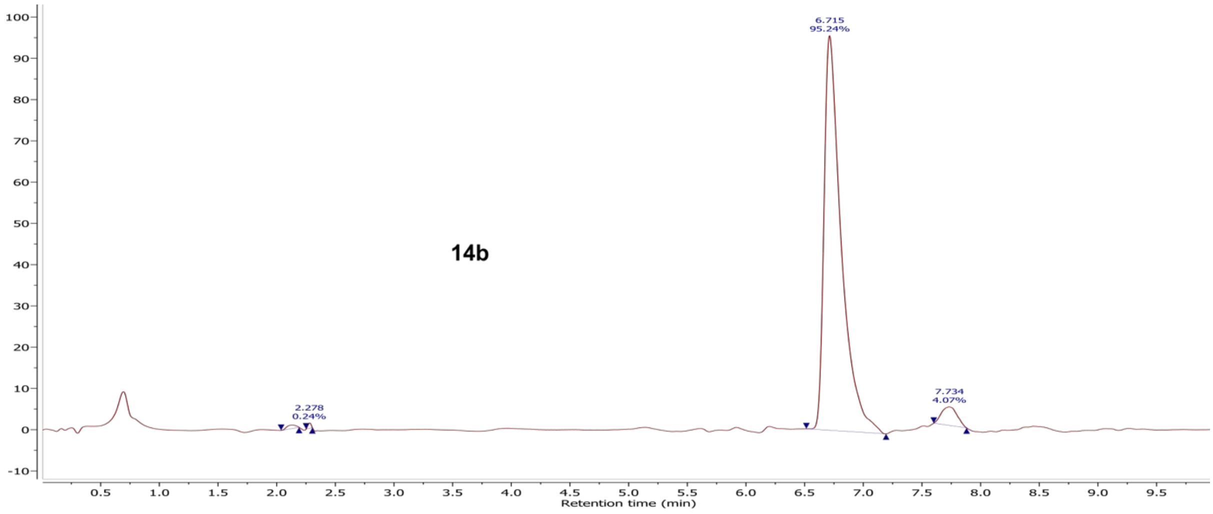

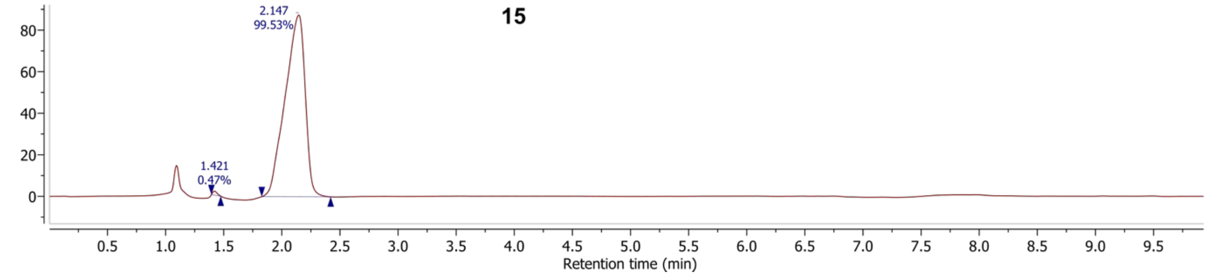

2 Supplementary References

(1) Holt-Martyn, J. P.; Chowdhury, R.; Tumber, A.; Yeh, T.-L.; Abboud, M. I.; Lippl, K.; Lohans, C. T.; Langley, G. W.; Figg Jr, W.; McDonough, M. A.; Pugh, C. W.; Ratcliffe, P. J.; Schofield, C. J. Structure-Activity Relationship and Crystallographic Studies on 4-Hydroxypyrimidine HIF Prolyl Hydroxylase Domain Inhibitors. *ChemMedChem* **2020**, *15*, 270–273.

(2) England, K. S.; Tumber, A.; Krojer, T.; Scozzafava, G.; Ng, S. S.; Daniel, M.; Szykowska, A.; Che, K.; von Delft, F.; Burgess-Brown, N. A.; Kawamura, A.; Schofield, C. J.; Brennan, P. E. Optimisation of a Triazolopyridine Based Histone Demethylase Inhibitor Yields a Potent and Selective KDM2A (FBXL11) Inhibitor. *Medchemcomm* **2014**, *5*, 1879–1886.

(3) Chen, B.; Ye, F.; Yu, L.; Jia, G.; Huang, X.; Zhang, X.; Peng, S.; Chen, K.; Wang, M.; Gong, S.; Zhang, R.; Yin, J.; Li, H.; Yang, Y.; Liu, H.; Zhang, J.; Zhang, H.; Zhang, A.; Jiang, H.; Luo, C.; Yang, C.-G. Development of Cell-Active N6-Methyladenosine RNA Demethylase FTO Inhibitor. *J. Am. Chem. Soc.* **2012**, *134*, 17963–17971.

(4) Huang, Y.; Yan, J.; Li, Q.; Li, J.; Gong, S.; Zhou, H.; Gan, J.; Jiang, H.; Jia, G.-F.; Luo, C.; Yang, C.-G. Meclofenamic Acid Selectively Inhibits FTO Demethylation of M6A over ALKBH5. *Nucleic Acids Res.* **2015**, *43*, 373–384.

(5) Zheng, G.; Cox, T.; Tribbey, L.; Wang, G. Z.; Iacoban, P.; Booher, M. E.; Gabriel, G. J.; Zhou, L.; Bae, N.; Rowles, J.; He, C.; Olsen, M. J. Synthesis of a FTO Inhibitor with Anticonvulsant Activity. *ACS Chem. Neurosci.* **2014**, *5*, 658–665.

(6) Wang, T.; Hong, T.; Huang, Y.; Su, H.; Wu, F.; Chen, Y.; Wei, L.; Huang, W.; Hua, X.; Xia, Y.; Xu, J.; Gan, J.; Yuan, B.; Feng, Y.; Zhang, X.; Yang, C.-G.; Zhou, X. Fluorescein Derivatives as Bifunctional Molecules for the Simultaneous Inhibiting and Labeling of FTO Protein. *J. Am. Chem. Soc.* **2015**, *137*, 13736–13739.

(7) Peng, S.; Xiao, W.; Ju, D.; Sun, B.; Hou, N.; Liu, Q.; Wang, Y.; Zhao, H.; Gao, C.; Zhang, S.; Cao, R.; Li, P.; Huang, H.; Ma, Y.; Wang, Y.; Lai, W.; Ma, Z.; Zhang, W.; Huang, S.; Wang, H.; Zhang, Z.; Zhao, L.; Cai, T.; Zhao, Y.-L.; Wang, F.; Nie, Y.; Zhi, G.; Yang, Y.-G.; Zhang, E. E.; Huang, N. Identification of Entacapone as a Chemical Inhibitor of FTO Mediating Metabolic Regulation through FOXO1. *Sci. Transl. Med.* **2019**, *11*, eaau7116.

(8) Qiao, Y.; Zhou, B.; Zhang, M.; Liu, W.; Han, Z.; Song, C.; Yu, W.; Yang, Q.; Wang, R.; Wang, S.; Shi, S.; Zhao, R.; Chai, J.; Chang, J. A Novel Inhibitor of the Obesity-Related Protein FTO. *Biochemistry* **2016**, *55*, 1516–1522.

(9) Aik, W.; Demetriades, M.; Hamdan, M. K. K.; Bagg, E. A. L.; Yeoh, K. K.; Lejeune, C.; Zhang, Z.; McDonough, M. A.; Schofield, C. J. Structural Basis for Inhibition of the Fat Mass and Obesity Associated Protein (FTO). *J. Med. Chem.* **2013**, *56*, 3680–3688.

(10) Rose, N. R.; McDonough, M. A.; King, O. N. F.; Kawamura, A.; Schofield, C. J. Inhibition of 2-Oxoglutarate Dependent Oxygenases. *Chem. Soc. Rev.* **2011**, *40*, 4364–4397.

(11) Wu, R.; Yao, Y.; Jiang, Q.; Cai, M.; Liu, Q.; Wang, Y.; Wang, X. Epigallocatechin Gallate Targets FTO and Inhibits Adipogenesis in an MRNA M6A-YTHDF2-Dependent Manner. *Int. J. Obes.* **2018**, *42*, 1378–1388.

(12) Zhang, L.; Ren, T.; Wang, Z.; Wang, R.; Chang, J. Comparative Study of the Binding of 3 Flavonoids to the Fat Mass and Obesity-Associated Protein by Spectroscopy and Molecular Modeling. *J. Mol. Recognit.* **2017**, *30*, e2606.

(13) Toh, J. D. W.; Sun, L.; Lau, L. Z. M.; Tan, J.; Low, J. J. A.; Tang, C. W. Q.; Cheong, E. J. Y.; Tan, M. J. H.; Chen, Y.; Hong, W.; Gao, Y.-G.; Woon, E. C. Y. A Strategy

Based on Nucleotide Specificity Leads to a Subfamily-Selective and Cell-Active Inhibitor of N6-Methyladenosine Demethylase FTO. *Chem. Sci.* **2015**, *6*, 112–122.
(14) Zhang, X.; Wei, L.-H.; Wang, Y.; Xiao, Y.; Liu, J.; Zhang, W.; Yan, N.; Amu, G.; Tang, X.; Zhang, L.; Jia, G. Structural Insights into FTO's Catalytic Mechanism for the Demethylation of Multiple RNA Substrates. *Proc. Natl. Acad. Sci.* **2019**, *116*, 2919–2924.
(15) Woon, E. C. Y.; Demetriades, M.; Bagg, E. A. L.; Aik, W.; Krylova, S. M.; Ma, J. H. Y.; Chan, M.; Walport, L. J.; Wegman, D. W.; Dack, K. N.; McDonough, M. A.; Krylov, S. N.; Schofield, C. J. Dynamic Combinatorial Mass Spectrometry Leads to Inhibitors of a 2-Oxoglutarate-Dependent Nucleic Acid Demethylase. *J. Med. Chem.* **2012**, *55*, 2173–2184.
